# S100β as a serum marker in endocrine resistant breast cancer

**DOI:** 10.1186/s12916-017-0836-2

**Published:** 2017-04-12

**Authors:** Sara Charmsaz, Éamon Hughes, Fiona T. Bane, Paul Tibbitts, Marie McIlroy, Christopher Byrne, Sinéad Cocchiglia, Jean McBryan, Bryan T. Hennessy, Róisín M. Dwyer, Michael J. Kerin, Arnold D. Hill, Leonie S. Young

**Affiliations:** 1 Endocrine Oncology Research Group, Department of Surgery, Royal College of Surgeons in Ireland, Dublin, Ireland; 2 Department of Surgery, Beaumont Hospital, Dublin, Ireland; 3 Department of Medical Oncology, Beaumont Hospital, Dublin, Ireland; 4 Department of Surgery, Lambe Institute for Translational Researcich, National University of Ireland Galway, Galway, Ireland

**Keywords:** Biomarker, Endocrine resistance, Breast cancer, S100β, Estrogen receptor

## Abstract

**Background:**

Endocrine therapy is standard treatment for estrogen receptor (ER)-positive breast cancer. However, its efficacy is limited by intrinsic and acquired resistance. Here the potential of S100β as a biomarker and inhibition of its signaling network as a therapeutic strategy in endocrine treated patients was investigated.

**Methods:**

The expression of S100β in tissue and serum was assessed by immunohistochemistry and an enzyme-linked immunosorbent assay, respectively. The S100β signaling network was investigated in cell line models of endocrine resistance by western blot, PCR, immunoprecipitation, and chromatin-immunoprecipitation. Endocrine resistant xenografts and tumor explants from patients with resistant tumors were treated with endocrine therapy in the presence and absence of the p-Src kinase inhibitor, dasatinib.

**Results:**

Tissue and serum levels of S100β were found to predict poor disease-free survival in endocrine-treated patients (*n* = 509, HR 2.32, 95% CI is 1.58–3.40, *p* < 0.0001 and *n* = 187, HR 4.009, 95% CI is 1.66–9.68, *p* = 0.002, respectively). Moreover, elevated levels of serum S100β detected during routine surveillance over the patient treatment period significantly associated with subsequent clinically confirmed disease recurrence (*p* = 0.019). In vivo studies demonstrated that endocrine treatment induced transcriptional regulation of S100β which was successfully disrupted with tyrosine kinase inhibition. In endocrine resistant xenografts and tumor explants from patients with endocrine resistant breast cancer, combined endocrine and dasatinib treatment reduced tumor proliferation and down-regulated S100β protein expression in comparison to endocrine treatment alone.

**Conclusions:**

S100β has potential as a new surveillance tool for patients with ER-positive breast cancer to monitor ongoing response to endocrine therapy. Moreover, endocrine resistant breast cancer patients with elevated S100β may benefit from combined endocrine and tyrosine-kinase inhibitor treatment.

**Trial registration:**

ClinicalTrials.gov, NCT01840293). Registered on 23 April 2013. Retrospectively registered.

**Electronic supplementary material:**

The online version of this article (doi:10.1186/s12916-017-0836-2) contains supplementary material, which is available to authorized users.

## Background

Endocrine therapies, both tamoxifen and aromatase inhibitors (AIs), successfully treat ER-positive breast cancer. A small but significant number of patients, however, will acquire resistance and develop disease recurrence [1]. Clinically, undetected resistant tumors continue to be exposed to adjuvant endocrine therapy, which can contribute to tumor progression and development of metastatic disease [1]. Despite concentrated efforts to discover robust biomarkers of endocrine sensitivity, no clinical assay to monitor real-time patient response to endocrine treatment has been developed. Understanding tumor adaptation to endocrine therapy has the potential to uncover new biomarkers of drug sensitivity and novel therapeutic targets to detect and treat endocrine resistant metastatic disease [2].

At a cellular level, increased tumor plasticity occurs in endocrine resistant breast cancer compared to sensitive tumors. Studies from our group and others suggest that enhanced tyrosine kinase signaling, re-expression of homeobox (HOX) developmental proteins along with activation of steroid receptor co-activator-1 (SRC-1) proteins cooperate to regulate breast cancer tumor adaptability [3–5]. HOXC11 and SRC-1 transcriptionally regulate the calcium-binding protein S100β, and overexpression of SRC-1 together with HOXC11 results in increased expression of S100β [3, 4, 6]. S100β is a member of the S100 family of calcium-binding proteins, several of which, including S100A7 and a truncated form of S100P, have been associated with progressive breast cancer [7–9]. S100β is detected at low levels in human serum; however, elevated levels of serum S100β have been reported following injuries to the central nervous system and in different types of tumors including melanoma and breast cancer [3, 10–12].

Endocrine resistance is marked by a shift from steroid-dependent to tyrosine kinase-dependent signaling [13]. Src is a non-receptor tyrosine kinase which has roles in tumor progression, epithelial-to-mesenchymal transition, angiogenesis, and the development of metastasis [14]. Interaction of SRC-1 and Src kinase has been described in endometrial cancer, suggesting the possibility of targeting SRC-1/HOXC11 interaction through inhibition of Src kinase activity [15]. Clinically, one of the most widely used Src kinase inhibitors is dasatinib, which is a small molecule inhibitor that targets kinase proteins including Src, BCR-ABL, cKit, and PDGFRβ [16]. Results from clinical trials evaluating dasatinib in patients with advanced breast cancer demonstrated efficacy in a subset of patients and suggests that patient stratification would significantly enhance the benefit of Src kinase inhibition [17, 18]. However, to date no gene signature has defined tumors clinically sensitive to dasatinib as a single agent [19].

Here we examine the prognostic potential of serum S100β as a monitoring tool for tumor response in patients with breast cancer on endocrine treatment. We investigate the mechanism of S100β production in endocrine resistant breast cancer and demonstrate that inhibition of the Src kinase pathway with the kinase inhibitor dasatinib can reduce tumor progression in vivo.

## Methods

### Ethics

All clinical materials and, where relevant, informed consent were collected following ethical approval from Beaumont Hospital Medical Research Ethics Committee, St. Vincent’s University Hospital Medical Research Ethics Committee, and Galway University Hospital Clinical Research Ethics Committee. This study was designed in accordance with the guidelines concerning tumor marker studies [20].

All mouse experiments were performed in accordance with the European Communities Council Directive (86/609/EEC) and were reviewed and approved by Research Ethics Committee under license from the Department of Health.

### Patients and study design

For prognostic tissue studies, formalin-fixed, paraffin-embedded (FFPE) breast tumor samples (*n* = 1079) were obtained from archival cases at St. Vincent’s University Hospital and Beaumont Hospital, Dublin, Ireland from 1987–1999 and from 2004–2008, respectively.

For prognostic serum studies, blood samples were collected from patients with breast cancer attending the National University Hospital Galway and Beaumont Hospital from 1993–2006 and 2008–2014, respectively, prior to initial surgery for the resection of primary breast tumor (*n* = 346).

For monitoring serum studies, pre-operative and serial post-operative samples were collected from breast cancer patients enrolled in a dual-center Cancer Trials Ireland-led trial (ICORG 09/07;NCT01840293/(ClinicalTrials.gov)) from 2008–2014 (*n* = 181).

**Fig. 1:**
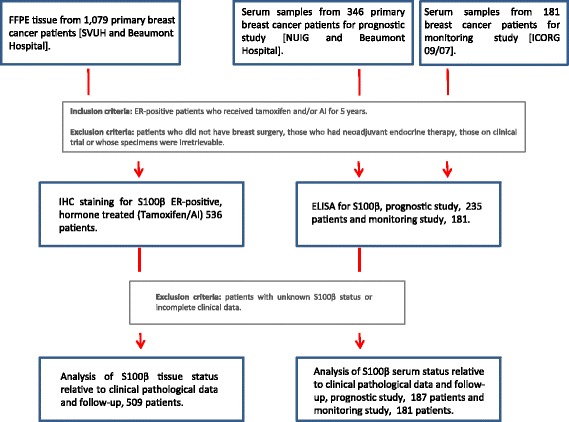
Flow chart of patients with breast cancer recruited to the clinical studies. For prognostic tissue studies, FFPE breast tumor samples (*n* = 1079) were obtained from archival cases at St. Vincent’s University Hospital and Beaumont Hospital, Dublin, Ireland from 1987–1999 and from 2004–2008, respectively. For prognostic serum studies, blood samples were collected from breast cancer patients attending the National University Hospital Galway and Beaumont Hospital from 1993–2006 and 2008–2014, respectively, prior to initial surgery for the resection of primary breast tumor (*n* = 346). For monitoring serum studies, pre-operative and serial post-operative samples were collected from patients with breast cancer enrolled in a multicenter Cancer Trials Ireland-led trial (ICORG 09/07;NCT01840293/(ClinicalTrials.gov)) from 2008–2014 (*n* = 181)

Included in this study were ER-positive patients who received tamoxifen and/or AI (*n* = 625, tissue and *n* = 235, serum) for 5 years, which was discontinued only in those who suffered a relapse. Excluded from the analysis were patients who did not have breast surgery, those who had neoadjuvant endocrine therapy, those on clinical trial or whose specimens were irretrievable, patients with unknown S100β status, or those with incomplete clinical data. Data on patients included pathological characteristics (tumor size, grade, lymph node status, ER/PR status) as well as treatment with radiotherapy/chemotherapy. Detailed follow-up data (median 6.4 years for tissue and 5.5 years for serum studies) were collected. The primary endpoint in this study was breast-specific disease-free survival. A flow diagram with patient inclusion and exclusion criteria for this study is presented in Fig. 1.

### Specimens

Breast cancer tissue samples were formalin-fixed and paraffin embedded (FFPE) and a tissue microarray (TMA) was constructed as previously described [21]. Serum samples were collected from age-matched healthy volunteers (*n* = 10, median age 63.3 years). Serum was prepared by centrifugation (400 g for 10 min) and stored within 3 h of collection at –80 °C.

Breast cancer ex vivo studies were carried out using fresh tumor samples. Samples were cultured on gelatin sponges (Spongostan, Johnson & Johnson Medical, Gargrave, UK) in Minimum Essential Medium (MEM)/10% fetal calf serum, l‐glutamine, 10^–6^ M letrozole with or without 1 μM dasatinib [22] and incubated for 5 days. Tumors were formalin-fixed and embedded.

### Assessment of tissue S100β

TMAs were immunostained using monoclonal mouse anti-S100β antibody (AB-14849, Abcam, Cambridge, UK) specific for S100β alpha-beta heterodimer and beta-beta homodimer. TMAs were scored using the Allred system as previously described [23]. A score of ≥3 was defined as positive. Two independent observers, without knowledge of prognostic factors, scored the slides. The kappa score for the agreement between the two independent reviewers was 0.935.

### Clinical assay for serum S100β

Serum S100β levels were determined using a commercial enzyme-linked immunosorbent assay (ELISA) kit (364701, Diasorin, Stillwater, MN, USA) according to the manufacturer’s instructions. The precision of the assay is >85%, and the detection limit is 0.03 μg/L.

S100β cut-off levels were determined in a training set comprising ten breast cancer patients with no relapse, ten patients who went on to have confirmed disease recurrence, and ten aged matched healthy controls. The upper limit of normal was calculated (mean + (t_0.975,n-1_ x √(n+1/n) x SD)), and 0.13 μg/L was taken as the cut-off (Additional file 1: Figure S1); outliers were calculated as √0.13 μg/L. A validation study was carried out in 76 patients with breast cancer and 59 ER-positive breast cancer patients; S100β serum status was analyzed in relation to patient pathologic characteristics and disease recurrence (Additional file 2: Table S1).

### Cell studies

The endocrine sensitive cell line (MCF-7) was obtained from the American Type Culture Collection (ATCC), and the endocrine resistant cell line (LY2) was a gift from Robert Clarke (Georgetown University, Washington, DC, USA) [24]. Cells were maintained as previously described [25]. AI resistant LetR cells and AI sensitive ARO cells were created as described previously [3]. Cell lines were tested (Source Biosciences, Life Biosciences, Nottingham, UK) for authenticity in accordance with ATCC guidelines.

### Xenograft studies

Mouse experiments were performed as previously described [26]. Endocrine sensitive (MCF-7) and endocrine resistant (LY2) xenograft models were established. Mice were supplemented with estrogen (0.25 mg/pellet, 60-day release) and treated with tamoxifen (5 mg/pellet, 60-day release; Innovative Research of America, Sarasota, FL, USA) and dasatinib (50 mg/kg/day, oral gavage; Bristol-Myers Squibb, New York, NY, USA) as indicated. Tumor growth was recorded twice weekly by caliper measurements, and tumors were imaged using an IVIS whole body imaging system (Xenogen Corp, Alameda, CA, USA) to detect luciferase activity of the LY2-luc cells. Metastasis was detected by shielding the primary tumor, and quantitative measurements of metastatic deposits were calculated using Living Image analysis software (Xenogen). Formation of metastasis to the organs was also confirmed by immunohistochemical analysis of the lung, liver, and bone. Blood was collected at 2-week intervals by facial vein bleeding to monitor S100β levels in serum.

### Chromatin immunoprecipitation

LY2 cells were treated with tamoxifen or tamoxifen and dasatinib. Chromatin immunoprecipitation (ChIP) was performed as previously described [23] using mouse anti-HOXC11 6 μg ((15-288-22000 F, Genway Biotech, San Diego, CA, USA)) and mouse anti-SRC-1 12 μg (F10, Santa Cruz Biotechnology, Dallas, TX, USA). S100β promoter-specific primers were used to amplify the DNA forward: TGGCAGAGGAGAGAAGCTC and reverse: TTCCTGAGCGTCCTCTTGG.

### Western blot

Protein was harvested from breast cancer cells, electrophoresed and immunoblotted with mouse anti-S100β (AB-14849, Abcam, 1 in 250), anti-ERα (sc-8002, Santa Cruz Biotechnology, 1 in 1000), anti-PR (sc-538, Santa Cruz Biotechnology, 1 in 1000), anti-p-Src 416 (2101S, Cell Signaling Technology, Danvers, MA, USA, 1 in 1000), anti-c-Src (2109S, Cell Signaling, 1 in 1000), anti-SRC-1 (sc-8995, Santa Cruz Biotechnology, 1 in 150), anti-HOXC11 (15-288-22000 F, Genway Biotech, 1 in 1000), and anti-β-actin (A1978, Sigma Aldrich, St. Louis, MO, USA, 1 in 7500) as described previously [25].

### Statistical analysis

Statistical analysis was performed using Prism (GraphPad, San Diego, CA, USA) and Stata software (StataCorp, College Station, TX, USA). Stata software was used to perform receiver operating characteristic (ROC) analyses. A 95% confidence interval (CI) was used to determine significance. Survival times between groups were compared using the Wilcoxon test adjusted for censored values. Multivariate analysis for S100β was carried out using Cox proportional hazards, modeled with node, grade, and HER2 status using the Breslow method for ties. Fisher’s exact test for two by two tables was used for comparison of categorical data. A two-sided Student’s *t* test was used for continuous variables. Values of *p* ≤ 0.05 were considered significant.

## Results

### Elevated levels of S100β associate with poor disease-free survival in patients with endocrine-treated breast cancer

**Fig. 2:**
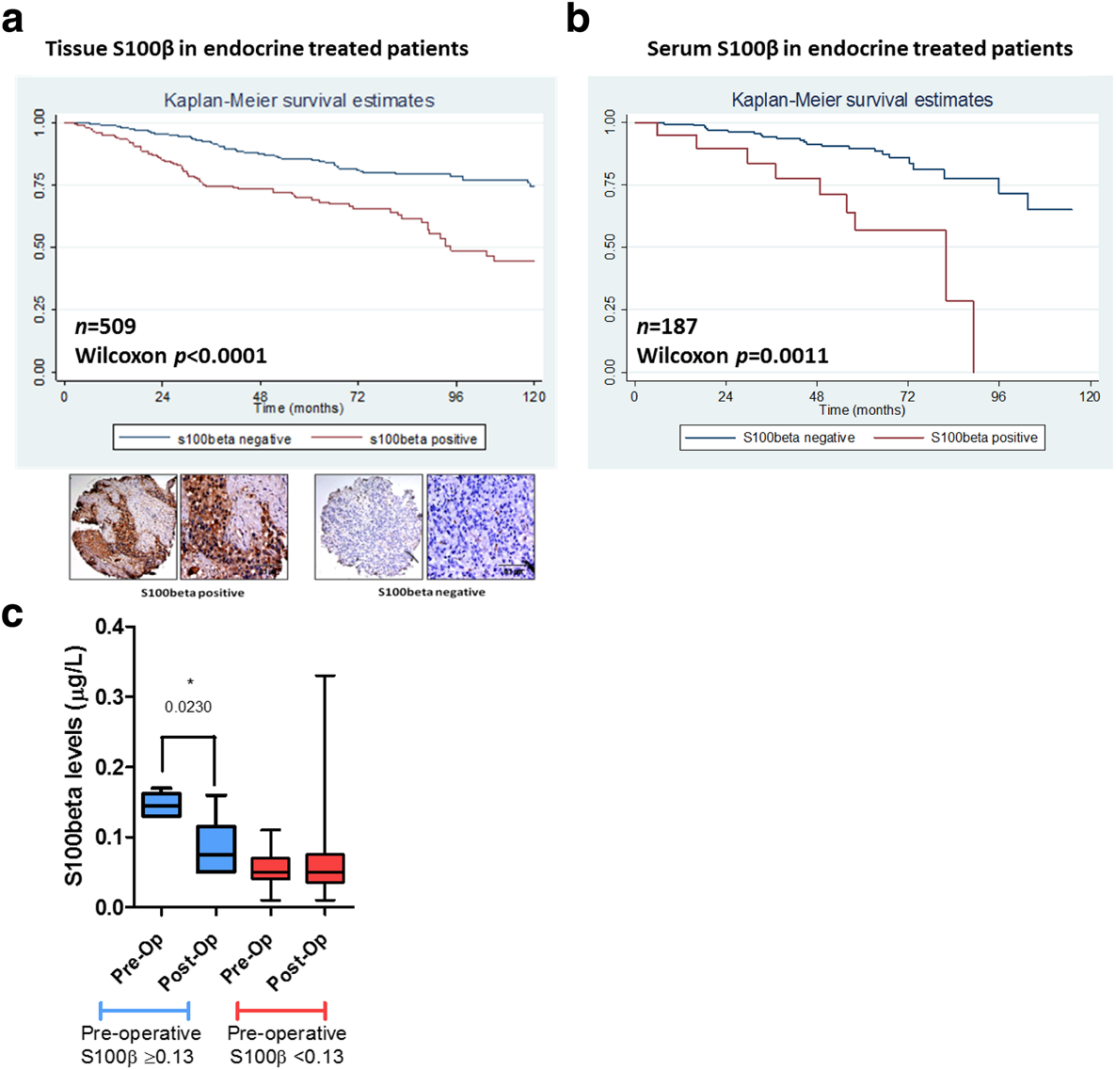
S100β associates with poor disease-free survival times in patients with endocrine-treated breast cancer. **a** S100β tissue expression strongly associated with poor disease-free survival in a TMA patient cohort of 509 ERα-positive, endocrine-treated patients as determined by Kaplan-Meier estimates of disease-free survival (HR 2.32, 95% CI is 1.58–3.40, *p* < 0.0001, Cox proportional hazard model). **b** 187 endocrine-treated ERα-positive patient serum samples were analyzed by ELISA for S100β using the established cut-off of 0.13 μg/L. S100β serum levels significantly associated with poor disease-free survival in the endocrine therapy-treated patient population (HR 3.09, 95% CI is 1.66–9.68, *p* = 0.002, Cox proportional hazard model). **c** Pre-operative elevated serum S100β returned to normal following surgical resection of the tumor in matched patient blood samples as measured by ELISA (55 individual patients, 6 pre-operative S100β ≥0.13 and 49 pre-operative S100β <0.13, paired *t* test error bars refer to 95% CI, *p* = 0.023)

The flow of the patients selected for the study is presented in Fig. 1. In FFPE tissue from adjuvant endocrine-treated patients expression of S100β strongly associated with incidence of recurrence (*n* = 536, *p* < 0.0001) (see Table 1) and poor disease-free survival time (*n* = 509, HR 2.32, 95% CI is 1.58–3.40, *p* < 0.0001, Cox proportional hazard model) (Fig. 2a). No association was observed between S100β and classic clinicopathological variables; however, as expected there was a strong association between S100β and HOXC11 expression (Table 1). As the possibility of assessing S100β levels in patient serum substantially enhances its clinical utility, a clinical assay suitable for use in patients with breast cancer was therefore developed. An appropriate cut-off of S100β serum levels in patients (0.13 μg/L) was established in a training set (Additional file 1: Figure S1) and verified in a validation study (*n* = 76, all breast cancer patients; *n* = 59, ER-positive breast cancer patients) (Additional file 2: Table S1). In our test endocrine-treated patient population, pre-operative elevated S100β in sera was also found to significantly predict disease-free survival on endocrine therapy (*n* = 187, HR 4.009, 95% CI is 1.66–9.68, *p* = 0.002, Cox proportional hazard model) (Fig. 2b, Table 1). S100β serum levels did not associate with any clinicopathological parameters with the exception of the progesterone receptor (PR). The S100β assay predicted disease recurrence in endocrine-treated patients with a specificity of 93% and a sensitivity of 27%.

**Table 1:** Associations of S100β expression in ER-positive endocrine-treated breast cancer tissue (*n* = 536) and serum (*n* = 187) with clinicopathologic variables and disease recurrence using Fisher’s exact test. Associations between disease-free survival times and individual variables using Wilcoxon. Median age for patient TMA and serum samples was 56.12 and 51.15 years respectively, and median follow-up time was 76.84 and 66.1 months respectively

Patient TMA						Patient serum samples					
Variable	Total *n*	S100β + ve	S100β –ve	*p* Fisher	*p* Wilcoxon		Total *n*	S100β ≥0.13 μg/L	S100β <0.13 μg/L	*p* Fisher	*p* Wilcoxon
S100β	536	34.7%	65.30%		<0.0001		187	10.16%	89.84%		0.0011
Age											
≥55	303	56.45%	56.57%	1.0	0.4		75	47.37%	39.29%	0.622	0.4
<55	233	43.55%	43.43%				112	52.63%	60.71%		
PR											
+ ve	321	65.50%	66.56%	0.841	0.4		159	57.89%	88.62%	0.002	0.8
− ve	164	34.50%	33.44%				27	42.11%	11.38%		
HER2											
+ ve	85	18.93%	17.10%	0.619	0.2		26	15.79%	13.69%	0.732	0.7
− ve	394	81.07%	82.90%				161	84.21%	86.31%		
Nodal status											
+ ve	273	49.46%	52.62%	0.524	<0.0001		99	53.33%	58.71%	0.786	0.05
− ve	257	50.54%	47.38%				71	46.67%	41.29%		
Grade											
≥ Grade 3	147	34.88%	27.10%	0.079	0.03		52	31.58%	27.38%	0.788	0.2
< Grade 3	346	65.12%	72.90%				135	68.42%	72.62%		
Tumor size											
≥20 mm	369	70.97%	67.71%	0.493	0.004		137	94.44%	86.33%	0.473	0.5
<20 mm	167	29.03%	32.29%				20	5.56%	13.67%		
Hormonal therapy											
Tamoxifen	459	83.87%	86.57%	0.438	0.4		110	42.11%	60.71%	0.143	0.7
AI	126	25.81%	22.29%	0.392	0.5		108	73.68%	55.95%	0.152	0.2
Other therapy											
Chemotherapy	166	34.64%	31.23%	0.431	0.1		119	61.11%	66.26%	0.794	0.2
Radiation	296	62.16%	52.01%	0.028	0.5		121	70.59%	74.66%	0.771	0.01
Herceptin	23	6.49%	3.14%	0.077	0.06		10	5.26%	5.36%	1.00	0.87
Recurrence											
+ ve	156	40.32%	23.14%	<0.0001			33	47.37%	14.29%	0.002	
− ve	380	59.68%	76.86%				154	52.63%	85.71%		
HOXC11											
+ ve	74	83.93%	18.49%	<0.0001	<0.0001						
− ve	128	16.07%	81.51%

Matched pre-operative and post-operative (<12 months) serum levels were measured in 55 patients. Surgical resection of the patient primary tumor returned elevated levels of serum S100β to normal (6 individual patients with elevated S100β, *p* = 0.023) (Fig. 2c, Additional file 2: Table S2). These data suggest that elevated levels of S100β can mark the presence of tumor burden in breast cancer patients and have the potential to predict disease progression in a subset of patients on endocrine treatment.

### Metastasis forms in the presence of tamoxifen in resistant breast cancer in vivo which is marked by S100β

**Fig. 3:**
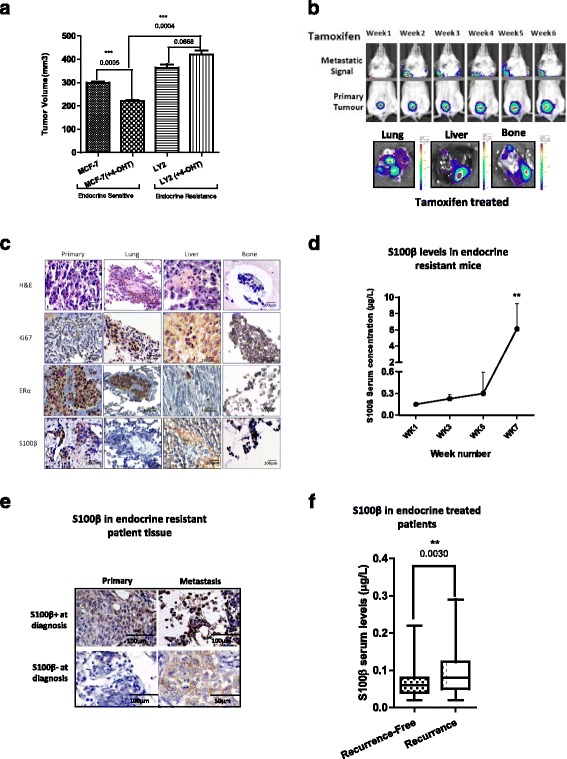
Metastasis forms in resistant breast cancer in vivo which is marked by S100β. **a** Endocrine sensitive MCF-7 and endocrine resistant LY2 cells were injected into the mammary fat pad of 6-week-old Balbc/SCID mice. Mice were treated with estrogen and with or without slow release tamoxifen pellets. Tumor volume was decreased significantly in the endocrine sensitive model with tamoxifen treatment (*p* = 0.0005, *n* = 3), and there was a small, but not significant, increase in tumor volume in the endocrine resistant model with tamoxifen treatment compared to the untreated endocrine resistant model (*p* = 0.068, *n* = 3). There was also a significant increase in tumor volume with tamoxifen treatment in the endocrine sensitive compared to endocrine resistant model (*p* = 0.0004, *n* = 3). **b** Metastasis was formed in the presence of tamoxifen treatment at week 2 which increased gradually until the experimental endpoint. Metastasis was visualized and quantified using in vivo and ex vivo IVIS imaging at the experimental endpoint (week 7). **c** Formation of metastasis in the organs was confirmed at experimental endpoint (week 7) by immunohistochemical (*IHC*) analysis. Representative images of primary tissue, lung, liver, and bone. H&E staining demonstrated the presence of tumor epithelial cells in the metastatic sites, and nuclear Ki67 indicated active proliferation. ERα expression was maintained throughout the experiment in the primary tumor and in the lungs, liver, and bone metastases and S100β expression in primary tissue, lung, liver, and bone. (Representative image *n* = 7.) **d** S100β levels in the serum of mice treated with tamoxifen increased concurrently with metastasis (non-parametric *t* test of blood between weeks 5 and 7, *p* = 0.0012). **e** In matched patients who recurred on endocrine therapy high expression of S100β in their primary tumor was maintained in metastatic tissue, while patients with low S100β in their primary tumor gained expression of S100β in their metastatic tissue at diagnosis as determined by IHC analysis (*n* = 4). **f** Post-operative monitoring levels of S100β was significantly higher in patients with metastatic disease (*n* = 96 Recurrence-Free and *n* = 22 Recurrence patients; error bars refer to 95% CI, *p* = 0.003)

Here we investigated alterations in tumor burden in endocrine sensitive versus resistant tumors in the presence and absence of treatment in an in vivo xenograft model. Tamoxifen-treated endocrine resistant models had a greater tumor volume in comparison to tamoxifen-treated endocrine sensitive models (*p* = 0.0004). A small increase in tumor volume was observed in the tamoxifen-treated endocrine resistant xenografts in comparison to the untreated endocrine resistant xenograft, though this was not significant (*p* = 0.0668) (Fig. 3a). The development of metastatic disease was observed in tamoxifen-treated endocrine resistant xenografts, and formations of micrometastatic deposits in the lung, liver, and bone were evident (Fig. 3b). Immunohistochemical (IHC) analysis of the primary and the matched metastatic tumors from the tamoxifen-treated endocrine resistant xenografts showed expression of ER, as well as the proliferation marker Ki67, in both the primary and metastatic tumors (Fig. 3c).

As we have previously reported, S100β is an output of a network activated by prolonged exposure to endocrine treatment [3]; therefore, it has the potential to act as a biomarker for the emergence of metastatic disease. Elevated S100β serum levels at week 7 post-implantation coincided with increased tumor burden and manifestation of distant micrometastasis (two-sided Student’s *t* test, *p* = 0.0012) (Fig. 3d). These observations were confirmed at the level of the tumor tissue where expression of S100β, though undetectable in the lung, was highly expressed in the liver and bone (Fig. 3c).

### Elevated S100β protein during the treatment period marks disease recurrence in endocrine-treated patients

In matched primary and metastatic tumors from patients who had recurred on endocrine therapy (*n* = 4), S100β was detected in all of the metastatic tumors even if it was absent from the primary tumor tissue (Fig. 3e and Additional file 2: Table S3).

In sequential serum samples taken during the treatment period, elevated levels of S100β were recorded in endocrine-treated patients with subsequent clinical and pathological disease progression (*n* = 118, *p* = 0.003) (Fig. 3f and Table 2). The S100β monitoring assay detected disease recurrence in endocrine-treated patients with a specificity of 95% and a sensitivity of 23%.

**Table 2:** Associations of clinicopathologic variables and disease outcome in ER-positive endocrine-treated breast cancer patients (*n* = 118) with S100β using Fisher’s exact test. Median age was 51.22 years , median follow-up time was 59.71 months and monitoring blood tests were taken >12 months post-operation (median 24.32 months)

	S100β ≥0.13 μg/L*n =* 10 (8.47%)	S100β <0.13 μg/L*n =* 108 (91.53%)	*p* value
Age			
≥55	3 (30%)	64 (59.26%)	0.099
< 55	7 (70%)	44 (40.74%)	
PR			
+ ve	8 (80%)	87 (80.56%)	1.00
–ve	2 (20%)	21 (19.44%)	
HER2			
+ ve	3 (30%)	52 (51.49%)	0.097
–ve	7 (70%)	49 (48.51%)	
Node			
+ ve	2 (22.22%)	43 (47.25%)	0.162
–ve	7 (77.78%)	48 (52.75%)	
Grade			
≥3	4 (40%)	26 (24.07%)	0.273
<3	6 (60%)	82 (75.93%)	
Size			
≥20 mm	7 (100%)	81 (96.43%)	1.00
< 20 mm	–	3 (3.57%)	
Recurrence			
+ ve	5 (50%)	17 (15.74%)	0.019 (*r* = +0.245)
–ve	5 (50%)	91 (84.26%)	

Patients with a positive S100β status who failed endocrine treatment had an enhanced risk of developing distant metastasis (79%) relative to local disease recurrence (21%). Sites of distant recurrence recorded included the bone, lung, and liver (Additional file 1: Figure S2).

### Endocrine treatment can induce transcriptional regulation of S100β which is disrupted by tyrosine kinase inhibition

**Fig. 4:**
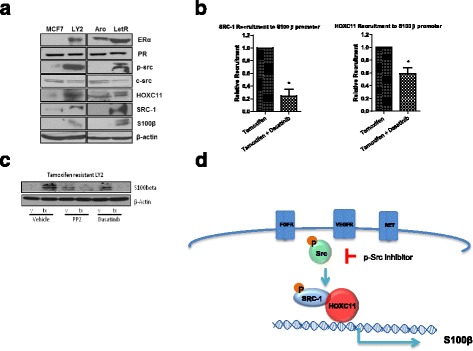
Endocrine treatment induces transcriptional regulation of S100β which is disrupted by tyrosine kinase inhibition. **a** Expression of ERα, PR, p-Src, c-Src, HOXC11, SRC-1, S100β, and β-actin in a panel of endocrine resistant (*LY2, LetR*) and sensitive (*MCF-7, ARO*) cell lines (representative blots from *n* = 3). **b** Chromatin immunoprecipitation of SRC-1 and HOXC11 of the S100β promoter region in LY2 cells treated with tamoxifen alone or in combination with dasatinib inhibits SRC-1 and HOXC11 recruitment to the S100β promoter (graphs are representative of the mean relative recruitment values relative to tamoxifen-treated controls, *n* = 3 ± SEM, *p* ≤ 0.05). **c** Inhibition of S100β protein expression with tyrosine kinase inhibitors PP2 or dasatinib in the endocrine resistant LY2 cells treated with tamoxifen (representative blots from *n* = 3). **d** Schematic representation of S100β pathway, showing long-term exposure to endocrine therapy induces expression of S100β in an Src-dependent manner

We used cell line models to investigate the mechanism of production of S100β in endocrine resistance. Elevated levels of ERα, HOXC11, and SRC-1 along with their transcriptional target S100β were observed in both tamoxifen resistant (LY2) and aromatase inhibitor (AI) resistant (LetR) cells; however, no alteration was observed in PR levels (Fig. 4a). Consistent with the enhanced growth factor crosstalk described in endocrine resistance, increased levels of p-AKT, p-ERK and in particular p-Src were also observed in endocrine resistant cells (Fig. 4a, Additional file 1: Figure S3A).

Elevated levels of p-Src driven by endocrine treatment and growth factors were reduced by the specific p-Src inhibitor PP2 and the Src-ABL inhibitor dasatinib (Additional file 1: Figure S3B). Treatment driven HOXC11 interactions with its coactivator SRC-1 and HOXC11/SRC-1 recruitment to the S100β promoter were disrupted by p-Src kinase inhibition (Fig. 4b, Additional file 1: Figure S3C). Furthermore, tamoxifen-induced transcript and protein levels of S100β were inhibited in the presence of PP2 and dasatinib in the treatment resistant LY2 cells (Fig. 4c, Additional file 1: Figure S3D). Taken together, these molecular studies provided us with a model of S100β production in endocrine resistant breast cancer where endocrine treatment can induce rapid tyrosine kinase signaling to induce HOXC11/SRC-1 transcriptional activation of S100β. This signaling network can be disrupted in the presence of the p-Src kinase inhibitors PP2 or dasatinib (Additional file 1: Figure S3D).

### Tyrosine kinase signaling inhibition can repress the production of S100β and impede tumor progression in endocrine resistant breast cancer

**Fig. 5:**
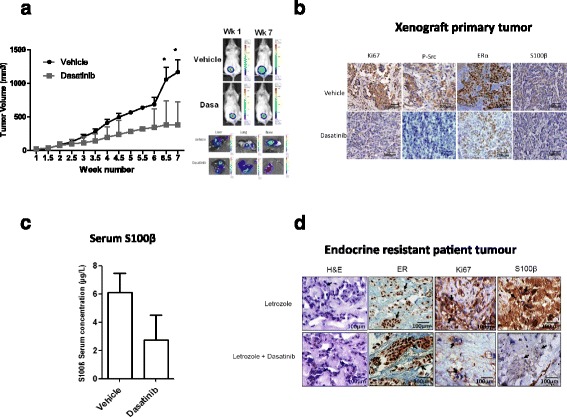
Combined endocrine and Src-kinase inhibitor treatment decreases tumor burden, which is marked by reduction in S100β. **a** In vivo model of endocrine resistant xenograft treated with dasatinib (50 mg/kg/day) in combination with tamoxifen showed a significant inhibition of tumor growth (mean tumor volume from caliper measurement, *n* = 7, error bars refer to 95% CI, *p* < 0.05). Representative IVIS images of mice at week 1 and week 7. Representative IVIS ex vivo images of tumor metastasis to lung, liver, and bone. **b** IHC analysis of tamoxifen-treated primary tumor from the endocrine resistant xenograft showed ERα expression and inhibition of Ki67, p-Src, SRC-1, HOXC11, and S100β expression after treatment with dasatinib. **c** Serum levels of S100β reduced in the xenograft mice treated with dasatinib at week 7 (*n* = 5, *p* = 0.085 one-tailed *t* test). **d** IHC revealed that dasatinib decreased expression of S100β and the proliferative marker Ki67 while maintaining the expression of ERα in an ex vivo explant model of tumor tissue from endocrine resistant patients after treatment with letrozole in the presence or absence of dasatinib (*n* = 2)

Functionally, inhibition of p-Src with dasatinib alone and in combination with endocrine therapy reduced cell proliferation in tamoxifen and AI resistant breast cancer cells (Additional file 1: Figure S4A). In endocrine resistant xenografts treated with tamoxifen, the addition of dasatinib therapy significantly reduced tumor volume in comparison to tamoxifen alone (two-sided Student’s *t* test, *p* < 0.05, Fig. 5a). Moreover, treatment with dasatinib increased cellular differentiation of the primary tumor and, in line with our in vitro proliferation studies, significantly reduced Ki67 expression (two-sided Student’s *t* test, *p* < 0.01, Additional file 1: Figure S4B).

In vivo p-Src inhibition reduced the endocrine-regulated signaling network. Dasatinib-treated mice maintained ERα status and reduced primary tumor expression of p-Src kinase, the co-activator SRC-1, the transcription factor HOXC11, and their target gene S100β (Fig. 5b, Additional file 1: Figure S5).

The ability of p-Src inhibition to affect endocrine therapy-induced metastatic progression was investigated. In cell line models of endocrine resistance and metastatic disease, dasatinib significantly reduced cell migration (Additional file 1: Figure S6A). Confirming a role for the adaptive signaling network in the migratory process, forced expression of the S100β transcriptional machinery (SRC-1 and HOXC11) significantly induced migration in endocrine sensitive cells, which was abolished with dasatinib treatment (Additional file 1: Figure S6B and C).

Consistent with the ability of S100β to mark the existence of disease progression in our patient population and in our xenograft model, treatment with dasatinib reduced circulating S100β levels in the xenografts compared to endocrine treatment alone (Fig. 5c). Furthermore, in breast cancer patients with clinically confirmed disease recurrence on AI therapy, ex vivo treatment of the resistant tumors with dasatinib in combination with letrozole maintained ERα expression, reduced Ki67, and switched off S100β expression in comparison to letrozole treatment alone (*n* = 2) (Fig. 5d and Additional file 2: Table S4). Together these data establish S100β as an output of the endocrine resistance network and support its use as a serum marker of the emergence of endocrine-related metastatic disease in breast cancer patients.

## Discussion

Early diagnosis of treatment resistant metastatic tumors will have a significant impact on the management of ER-positive patients who have failed endocrine treatment. To date gene signature assays of the primary tumor, such as EndoPredict and the PAM50 assay, can inform adjuvant treatment strategies [27–29]. However, these prognostic assays may have limited capability to take into consideration the ability of the tumor to adapt to its therapeutic environment or to monitor real-time patient treatment response.

The importance of tumor molecular heterogeneity in determining response to targeted therapy is now firmly established [30]. Furthermore, the ability of a subset of breast tumors to alter their molecular profile between primary and recurrent tumors is the subject of intense investigation [26, 31]. This tumor adaptability is controlled, at least in part, through transcription factors including HOX family members responding to the therapeutic environment [3, 32, 33].

Here we describe a mechanistically based serum biomarker, S100β, which is an output of an adaptability signaling network known to be important in the development of endocrine resistance [3]. Both tissue and serum S100β protein can predict disease-free survival in ER-positive endocrine-treated patients. No association was observed between either tissue or serum S100β and classic clinicopathological parameters, with the exception of a strong association between S100β serum and PR, which is consistent with the proposed role for PR and enhanced kinase signaling [34].

The ELISA-based S100β assay has a sensitivity of 27%, which is reflective of its ability to identify a subset of patients who subsequently have disease relapse. However, the specificity of the assay is high at 93%, indicating that the assay is accurate. The clinical value of this assay is that it can successfully detect patients who will not relapse [35]. Moreover, in this study monitoring S100β serum levels during the treatment period identified patients with subsequent clinical and pathological disease recurrence with a specificity of 95% and a sensitivity of 23%. These serum studies are somewhat limited due to the relatively low number of patients, which reflects the challenges in collecting serial serum samples from endocrine-treated breast cancer patients. The data presented in this study suggest that a large multicenter clinical trial to evaluate the efficacy of serum S100β for routine surveillance of ER-positive patients during their endocrine treatment period is now warranted.

Known growth factor/endocrine signaling crosstalk in resistant breast cancer makes targeting kinase pathways an attractive management strategy [13]. The data presented in this study suggest that a select number of endocrine resistant patients would benefit from kinase inhibition. In endocrine resistant cell lines and in vivo models, endocrine treatment activated kinase-dependent adaptability signaling networks and drove tumor cell growth and migration. Inhibition of kinase signaling with the pan-Src-ABL inhibitor dasatinib turned off the adaptability network, restored endocrine sensitivity, and retarded disease progression. Of note, reduced tumor aggression was marked by a decrease in S100β protein in both in vivo endocrine resistant models and in ex vivo tumors from patients with endocrine resistant breast cancer. Therefore, breast cancer patients with elevated S100β may benefit from combined endocrine and kinase inhibitor treatment.

## Conclusions

In conclusion, identification of the adaptability network with S100β has the potential to provide new information regarding the response of the patient tumor to ongoing endocrine therapy and act as a companion diagnostic to increase the efficacy of combined tyrosine kinase inhibitor treatment.

## Additional files

Additional file 1: Figure S1. Training set to establish S100β elevated cut-off. Serum S100β levels were determined using a commercial ELISA kit (Diasorin) according to the manufacturer’s instructions. To determine an appropriate S100β cut-off level, a training set was constructed comprising ten breast cancer patients with no relapse (*No Recurrence*), ten patients who went on to have confirmed disease recurrence (*Recurrence*), and ten aged matched controls (*Normal*). The upper limit of normal was calculated (mean + (t_0.975,n-1_ x √(n+1/n) x SD)), 0.13 μg/L was considered the upper end of normal and was taken as the cut-off. Patient clinicopathological parameters and treatment details are provided for No Recurrence and Recurrence patients. Median age of control patients is 62.8 years. **Figure S2.** Site of recurrence in patients with elevated S100β. Site of recurrence in patients with elevated pre-operative or monitoring serum S100β levels (*n* = 13). **Figure S3.** Dasatinib successfully inhibits p-Src expression in endocrine resistant breast cancer. (A) Expression of p-AKT, AKT, P-ERK, and ERK in panel of endocrine resistant (*LY2, LetR*) and sensitive (*MCF-7, ARO*) cell lines. (B) Tamoxifen resistant cells LY2 expressed increased levels of p-Src when treated with tamoxifen which was inhibited with PP2 (10 μM) and dasatinib (0.1 μM). Letrozole resistant cells LetR expressed increased levels of p-Src when treated with EGF which was inhibited with PP2 (10 μM) and dasatinib (0.1 μM). (C) Interactions between SRC-1 and the transcription factor HOXC11 were increased by 4-OHT (1 × 10^–7^ M) in tamoxifen resistant LY2 cells and by EGF (10 ng/ml) in letrozole resistant LetR cells. These interactions were inhibited in both cell lines by pre-treatment with the p-SRC inhibitors PP2 (10 μM) and dasatinib (0.1 μM). (D) mRNA expression of the HOXC11/SRC-1 complex’s downstream target gene S100β increased with tamoxifen (*T*) treatment compared with vehicle (*V*). This tamoxifen-induced expression was successfully inhibited when treated with dasatinib (*D*). **Figure S4.** Proliferation in resistant breast cancer cell lines following dasatinib treatment. (A) Dasatinib treatment (1 μM) for 72 h inhibits both estrogen- and tamoxifen-driven proliferation of the tamoxifen resistant cell line LY2 and AI resistant LetR measured by MTS assay (*n* = 3). (B) Expression of nuclear Ki67 significantly decreased in the dasatinib-treated xenograft primary tumors (*p* = 0.01). Studying the architecture of the epithelial cells in the primary tumors by H&E staining revealed that dasatinib-treated tumors had a more organized cell population than that of the controls. **Figure S5.** SRC-1 and HOXC11 scoring of primary tumors from endocrine resistant xenograft model. Primary tumors from vehicle- and dasatinib-treated mice were stained for SRC-1 and HOXC11 and analyzed for total positivity using Aperio Imagescope software. Results are expressed as mean protein positivity ± SEM, *p* ≤ 0.05. The expression of SRC-1 and HOXC11 was decreased in the dasatinib-treated group compared to that of the vehicle-treated group. **Figure S6.** Migratory potential which can be inhibited by dasatinib. (A) The migratory potential of endocrine resistant cell lines (LY2 and LetR) is elevated in comparison to that of their parental, the non-migratory cell line MCF-7. This migration was comparable to that of the highly metastatic triple negative breast cancer cell line MDA-MB-231. Treatment with dasatinib significantly inhibited this migration (*p* < 0.05). (B) Endocrine sensitive MCF-7 cells were stably transfected with SRC-1 and HOXC11 vectors to assay the effects of these proteins on migratory potential. The successful overexpression was confirmed by western blot (*n* = 3). (C) Overexpression of the transcription factor HOXC11 and SRC-1 significantly increased the migration of MCF-7 compared to the empty vector transfected cells (*p* < 0.05). Dasatinib treatment (1 μM) significantly inhibited both HOXC11- and SRC-1-driven cellular migration (*p* < 0.001). (PPTX 670 kb)

Additional file 2: Table S1. (A) S100β validation set (*n* = 76 ER-positive and ER-negative patients). (B) S100β validation set (*n* = 59 ER-positive patients). Association of S100β status with clinicopathological variables and disease recurrence using Fisher’s exact test. **Table S2.** Pre-operative and post-operative S100β serum levels in 55 ER-positive patients. Association of S100β status with clinicopathological variables using Fisher’s exact test. **Table S3.** S100β tissue expression in matched primary and metastatic tissue from ER-positive patients. **Table S4.** Patient details from explant study. Explant endocrine resistant tumor tissue (*n* = 2) was treated with AI therapy (letrozole) in the presence and absence of dasatinib. (PPTX 93 kb)

## Abbreviations

AI: Aromatase inhibitor
ChIP: Chromatin immunoprecipitation
ELISA: Enzyme-linked immunosorbent assay
ER: Estrogen receptor
FFPE: Formalin-fixed, paraffin embedded
HOX: Homeobox
HR: Hazard ratio
PCR: Polymerase chain reaction
PR: Progesterone receptor
SD: Standard deviation
SRC-1: Steroid receptor co-activator-1
TMA: Tissue microarray

## References

[1] Doughty JC (2008). A review of the BIG results: the Breast International Group 1-98 trial analyses. Breast.

[2] Johnston SR. Enhancing endocrine therapy for hormone receptor-positive advanced breast cancer: cotargeting signaling pathways. J Natl Cancer Inst. 2015;107(10). doi: https://doi.org/10.1093/jnci/djv212. 10.1093/jnci/djv212 26251289

[3] McIlroy M, McCartan D, Early S, O Gaora P, Pennington S, Hill AD, Young LS (2010). Interaction of developmental transcription factor HOXC11 with steroid receptor coactivator SRC-1 mediates resistance to endocrine therapy in breast cancer. Cancer Res.

[4] deBlacam C, Byrne C, Hughes E, McIlroy M, Bane F, Hill AD, Young LS (2011). HOXC11-SRC-1 regulation of S100beta in cutaneous melanoma: new targets for the kinase inhibitor dasatinib. Br J Cancer.

[5] Walsh CA, Qin L, Tien J, Young LS, Xu J (2012). The function of steroid receptor coactivator-1 in normal tissues and cancer. Int J Biol Sci.

[6] Zhang X, Hamada J, Nishimoto A, Takahashi Y, Murai T, Tada M, Moriuchi T (2007). HOXC6 and HOXC11 increase transcription of S100beta gene in BrdU-induced in vitro differentiation of GOTO neuroblastoma cells into Schwannian cells. J Cell Mol Med.

[7] Jiang WG, Watkins G, Douglas-Jones A, Mansel RE (2004). Psoriasin is aberrantly expressed in human breast cancer and is related to clinical outcomes. Int J Oncol.

[8] Zhao H, Wilkie T, Deol Y, Sneh A, Ganju A, Basree M, Nasser MW, Ganju RK (2015). miR-29b defines the pro-/anti-proliferative effects of S100A7 in breast cancer. Mol Cancer.

[9] Chung L, Phillips L, Lin MZ, Moore K, Marsh DJ, Boyle FM, Baxter RC (2015). A novel truncated form of S100P predicts disease-free survival in patients with lymph node positive breast cancer. Cancer Lett.

[10] Gebhardt C, Lichtenberger R, Utikal J (2016). Biomarker value and pitfalls of serum S100B in the follow-up of high-risk melanoma patients. J Dtsch Dermatol Ges.

[11] Sedaghat F, Notopoulos A (2008). S100 protein family and its application in clinical practice. Hippokratia.

[12] Donato R, Cannon BR, Sorci G, Riuzzi F, Hsu K, Weber DJ, Geczy CL (2013). Functions of S100 proteins. Curr Mol Med.

[13] Massarweh S, Schiff R (2006). Resistance to endocrine therapy in breast cancer: exploiting estrogen receptor/growth factor signaling crosstalk. Endocr Relat Cancer.

[14] Sen B, Johnson FM (2011). Regulation of SRC family kinases in human cancers. J Signal Transduct.

[15] Shah YM, Rowan BG (2005). The Src kinase pathway promotes tamoxifen agonist action in Ishikawa endometrial cells through phosphorylation-dependent stabilization of estrogen receptor (alpha) promoter interaction and elevated steroid receptor coactivator 1 activity. Mol Endocrinol.

[16] Eustace AJ, Crown J, Clynes M, O’Donovan N (2008). Preclinical evaluation of dasatinib, a potent Src kinase inhibitor, in melanoma cell lines. J Transl Med.

[17] Finn RS, Bengala C, Ibrahim N, Roche H, Sparano J, Strauss LC, Fairchild J, Sy O, Goldstein LJ (2011). Dasatinib as a single agent in triple-negative breast cancer: results of an open-label phase 2 study. Clin Cancer Res.

[18] Mayer EL, Baurain JF, Sparano J, Strauss L, Campone M, Fumoleau P, Rugo H, Awada A, Sy O, Llombart-Cussac A (2011). A phase 2 trial of dasatinib in patients with advanced HER2-positive and/or hormone receptor-positive breast cancer. Clin Cancer Res.

[19] Pusztai L, Moulder S, Altan M, Kwiatkowski D, Valero V, Ueno NT, Esteva FJ, Avritscher R, Qi Y, Strauss L (2014). Gene signature-guided dasatinib therapy in metastatic breast cancer. Clin Cancer Res.

[20] McShane LM, Altman DG, Sauerbrei W, Taube SE, Gion M, Clark GM (2005). Statistics Subcommittee of the NCIEWGoCD: REporting recommendations for tumour MARKer prognostic studies (REMARK). Br J Cancer.

[21] Dillon MF, Stafford AT, Kelly G, Redmond AM, McIlroy M, Crotty TB, McDermott E, Hill AD, Young LS (2008). Cyclooxygenase-2 predicts adverse effects of tamoxifen: a possible mechanism of role for nuclear HER2 in breast cancer patients. Endocr Relat Cancer.

[22] Centenera MM, Gillis JL, Hanson AR, Jindal S, Taylor RA, Risbridger GP, Sutherland PD, Scher HI, Raj GV, Knudsen KE (2012). Evidence for efficacy of new Hsp90 inhibitors revealed by ex vivo culture of human prostate tumors. Clin Cancer Res.

[23] McCartan D, Bolger JC, Fagan A, Byrne C, Hao Y, Qin L, McIlroy M, Xu J, Hill AD, Gaora P (2012). Global characterization of the SRC-1 transcriptome identifies ADAM22 as an ER-independent mediator of endocrine-resistant breast cancer. Cancer Res.

[24] Bronzert DA, Greene GL, Lippman ME (1985). Selection and characterization of a breast cancer cell line resistant to the antiestrogen LY 117018. Endocrinology.

[25] Redmond AM, Bane FT, Stafford AT, McIlroy M, Dillon MF, Crotty TB, Hill AD, Young LS (2009). Coassociation of estrogen receptor and p160 proteins predicts resistance to endocrine treatment; SRC-1 is an independent predictor of breast cancer recurrence. Clin Cancer Res.

[26] McBryan J, Fagan A, McCartan D, Bane FT, Vareslija D, Cocchiglia S, Byrne C, Bolger J, McIlroy M, Hudson L (2015). Transcriptomic profiling of sequential tumors from breast cancer patients provides a global view of metastatic expression changes following endocrine therapy. Clin Cancer Res.

[27] Filipits M, Rudas M, Jakesz R, Dubsky P, Fitzal F, Singer CF, Dietze O, Greil R, Jelen A, Sevelda P (2011). A new molecular predictor of distant recurrence in ER-positive, HER2-negative breast cancer adds independent information to conventional clinical risk factors. Clin Cancer Res.

[28] Parker JS, Mullins M, Cheang MC, Leung S, Voduc D, Vickery T, Davies S, Fauron C, He X, Hu Z (2009). Supervised risk predictor of breast cancer based on intrinsic subtypes. J Clin Oncol.

[29] Nielsen TO, Parker JS, Leung S, Voduc D, Ebbert M, Vickery T, Davies SR, Snider J, Stijleman IJ, Reed J (2010). A comparison of PAM50 intrinsic subtyping with immunohistochemistry and clinical prognostic factors in tamoxifen-treated estrogen receptor-positive breast cancer. Clin Cancer Res.

[30] Koren S, Bentires-Alj M (2015). Breast tumor heterogeneity: source of fitness, hurdle for therapy. Mol Cell.

[31] Saunus JM, Quinn MC, Patch AM, Pearson JV, Bailey PJ, Nones K, McCart Reed AE, Miller D, Wilson PJ, Al-Ejeh F (2015). Integrated genomic and transcriptomic analysis of human brain metastases identifies alterations of potential clinical significance. J Pathol.

[32] Blessing EM, Steenkamp MM, Manzanares J, Marmar CR (2015). Cannabidiol as a potential treatment for anxiety disorders. Neurotherapeutics.

[33] Pathiraja TN, Nayak SR, Xi Y, Jiang S, Garee JP, Edwards DP, Lee AV, Chen J, Shea MJ, Santen RJ (2014). Epigenetic reprogramming of HOXC10 in endocrine-resistant breast cancer. Sci Transl Med.

[34] Daniel AR, Hagan CR, Lange CA (2011). Progesterone receptor action: defining a role in breast cancer. Expert Rev Endocrinol Metab.

[35] Ankerst DP, Thompson IM (2006). Sensitivity and specificity of prostate-specific antigen for prostate cancer detection with high rates of biopsy verification. Arch Ital Urol Androl.

