# Supplementary material for: S100β as a serum marker in endocrine resistant breast cancer
Source: BMC Med. 2017 Apr 12;15:79. doi: 10.1186/s12916-017-0836-2 (PMC5389184; doi:10.1186/s12916-017-0836-2)
Supplement: Supplementary file 1 — Training set to establish S100β elevated cut-off. Serum S100β levels were determined using a commercial ELISA kit (Diasorin) according to the manufacturer’s instructions. To determine an appropriate S100β cut-off level, a training set was constructed comprising ten breast cancer patients with no relapse (No Recurrence), ten patients who went on to have confirmed disease recurrence (Recurrence), and ten aged matched controls (Normal). The upper limit of normal was calculated (mean + (t0.975,n-1 x √(n+1/n) x SD)), 0.13 μg/L was considered the upper end of normal and was taken as the cut-off. Patient clinicopathological parameters and treatment details are provided for No Recurrence and Recurrence patients. Median age of control patients is 62.8 years. Figure S2. Site of recurrence in patients with elevated S100β. Site of recurrence in patients with elevated pre-operative or monitoring serum S100β levels (n = 13). Figure S3. Dasatinib successfully inhibits p-Src expression in endocrine resistant breast cancer. (A) Expression of p-AKT, AKT, P-ERK, and ERK in panel of endocrine resistant (LY2, LetR) and sensitive (MCF-7, ARO) cell lines. (B) Tamoxifen resistant cells LY2 expressed increased levels of p-Src when treated with tamoxifen which was inhibited with PP2 (10 μM) and dasatinib (0.1 μM). Letrozole resistant cells LetR expressed increased levels of p-Src when treated with EGF which was inhibited with PP2 (10 μM) and dasatinib (0.1 μM). (C) Interactions between SRC-1 and the transcription factor HOXC11 were increased by 4-OHT (1 × 10^–7^ M) in tamoxifen resistant LY2 cells and by EGF (10 ng/ml) in letrozole resistant LetR cells. These interactions were inhibited in both cell lines by pre-treatment with the p-SRC inhibitors PP2 (10 μM) and dasatinib (0.1 μM). (D) mRNA expression of the HOXC11/SRC-1 complex’s downstream target gene S100β increased with tamoxifen (T) treatment compared with vehicle (V). This tamoxifen-induced expression was successfully inhibited when tre [file 12916_2017_836_MOESM1_ESM.pptx]

## Slide 1
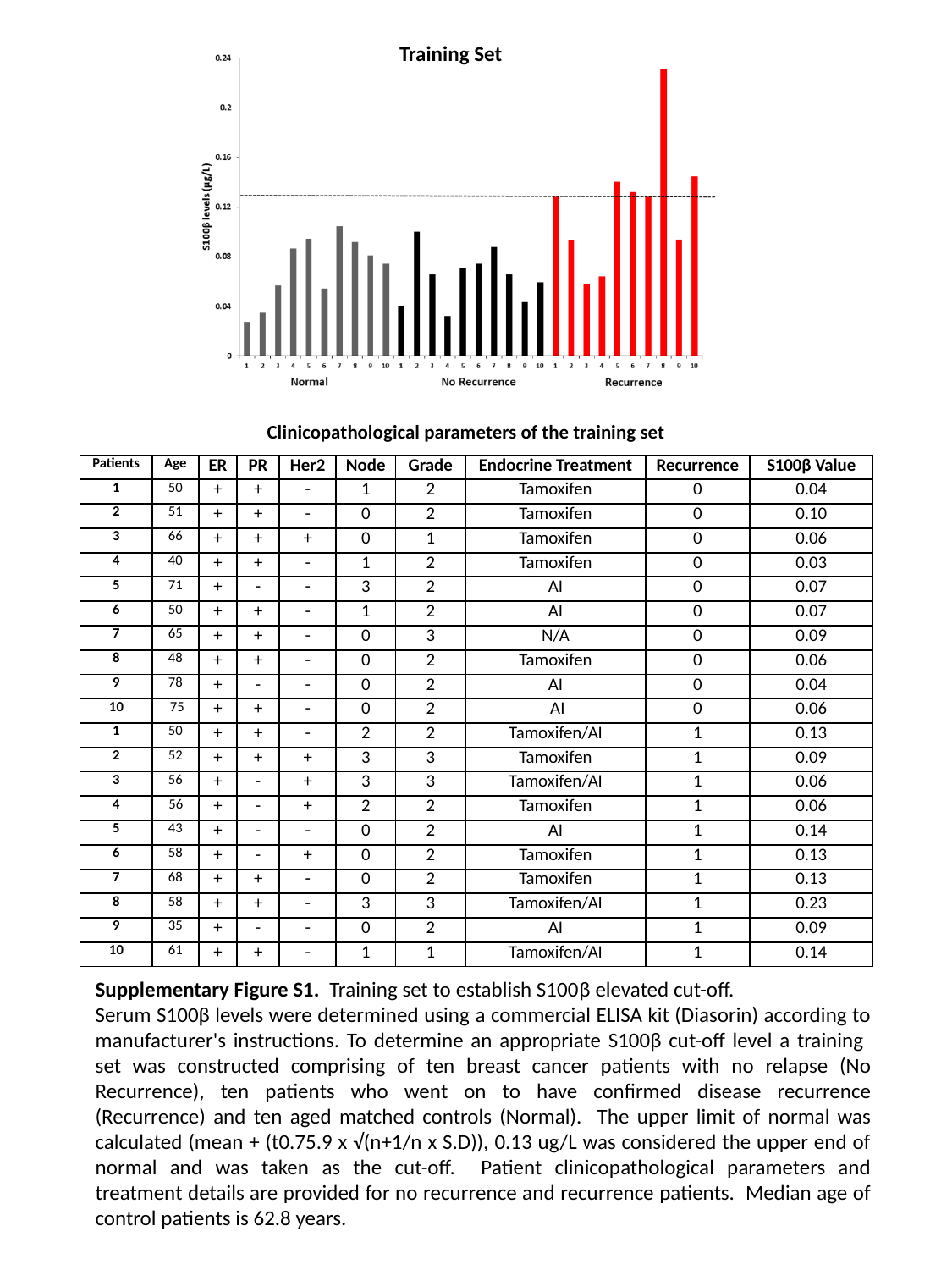

Training Set
Clinicopathological parameters of the training set
| Patients | Age | ER | PR | Her2 | Node | Grade | Endocrine Treatment | Recurrence | S100β Value |
| --- | --- | --- | --- | --- | --- | --- | --- | --- | --- |
| 1 | 50 | + | + | - | 1 | 2 | Tamoxifen | 0 | 0.04 |
| 2 | 51 | + | + | - | 0 | 2 | Tamoxifen | 0 | 0.10 |
| 3 | 66 | + | + | + | 0 | 1 | Tamoxifen | 0 | 0.06 |
| 4 | 40 | + | + | - | 1 | 2 | Tamoxifen | 0 | 0.03 |
| 5 | 71 | + | - | - | 3 | 2 | AI | 0 | 0.07 |
| 6 | 50 | + | + | - | 1 | 2 | AI | 0 | 0.07 |
| 7 | 65 | + | + | - | 0 | 3 | N/A | 0 | 0.09 |
| 8 | 48 | + | + | - | 0 | 2 | Tamoxifen | 0 | 0.06 |
| 9 | 78 | + | - | - | 0 | 2 | AI | 0 | 0.04 |
| 10 | 75 | + | + | - | 0 | 2 | AI | 0 | 0.06 |
| 1 | 50 | + | + | - | 2 | 2 | Tamoxifen/AI | 1 | 0.13 |
| 2 | 52 | + | + | + | 3 | 3 | Tamoxifen | 1 | 0.09 |
| 3 | 56 | + | - | + | 3 | 3 | Tamoxifen/AI | 1 | 0.06 |
| 4 | 56 | + | - | + | 2 | 2 | Tamoxifen | 1 | 0.06 |
| 5 | 43 | + | - | - | 0 | 2 | AI | 1 | 0.14 |
| 6 | 58 | + | - | + | 0 | 2 | Tamoxifen | 1 | 0.13 |
| 7 | 68 | + | + | - | 0 | 2 | Tamoxifen | 1 | 0.13 |
| 8 | 58 | + | + | - | 3 | 3 | Tamoxifen/AI | 1 | 0.23 |
| 9 | 35 | + | - | - | 0 | 2 | AI | 1 | 0.09 |
| 10 | 61 | + | + | - | 1 | 1 | Tamoxifen/AI | 1 | 0.14 |
Supplementary Figure S1. Training set to establish S100β elevated cut-off.
Serum S100β levels were determined using a commercial ELISA kit (Diasorin) according to manufacturer's instructions. To determine an appropriate S100β cut-off level a training set was constructed comprising of ten breast cancer patients with no relapse (No Recurrence), ten patients who went on to have confirmed disease recurrence (Recurrence) and ten aged matched controls (Normal). The upper limit of normal was calculated (mean + (t0.75.9 x √(n+1/n x S.D)), 0.13 ug/L was considered the upper end of normal and was taken as the cut-off. Patient clinicopathological parameters and treatment details are provided for no recurrence and recurrence patients. Median age of control patients is 62.8 years.

## Slide 2
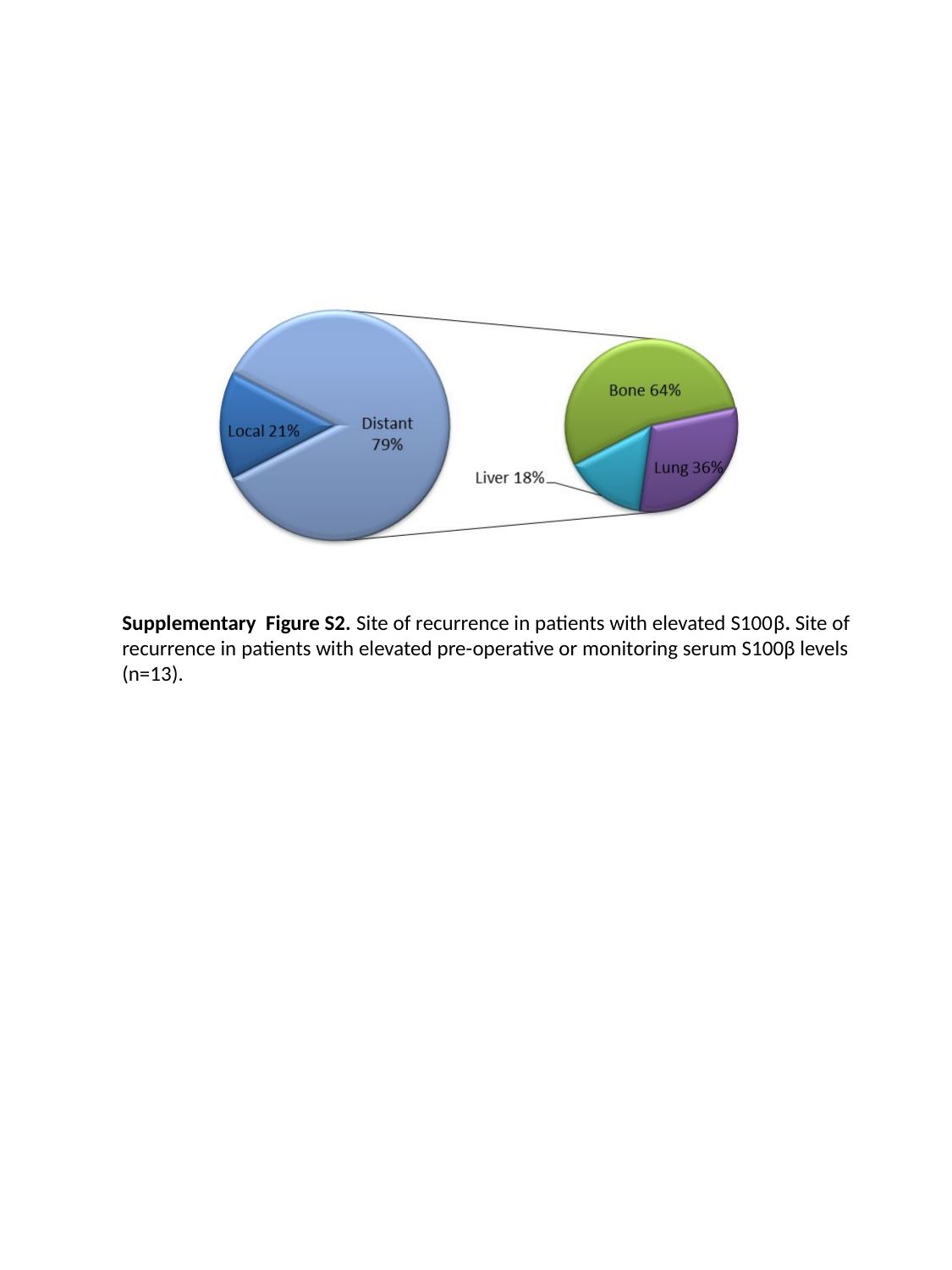

Supplementary Figure S2. Site of recurrence in patients with elevated S100β. Site of recurrence in patients with elevated pre-operative or monitoring serum S100β levels (n=13).

## Slide 3
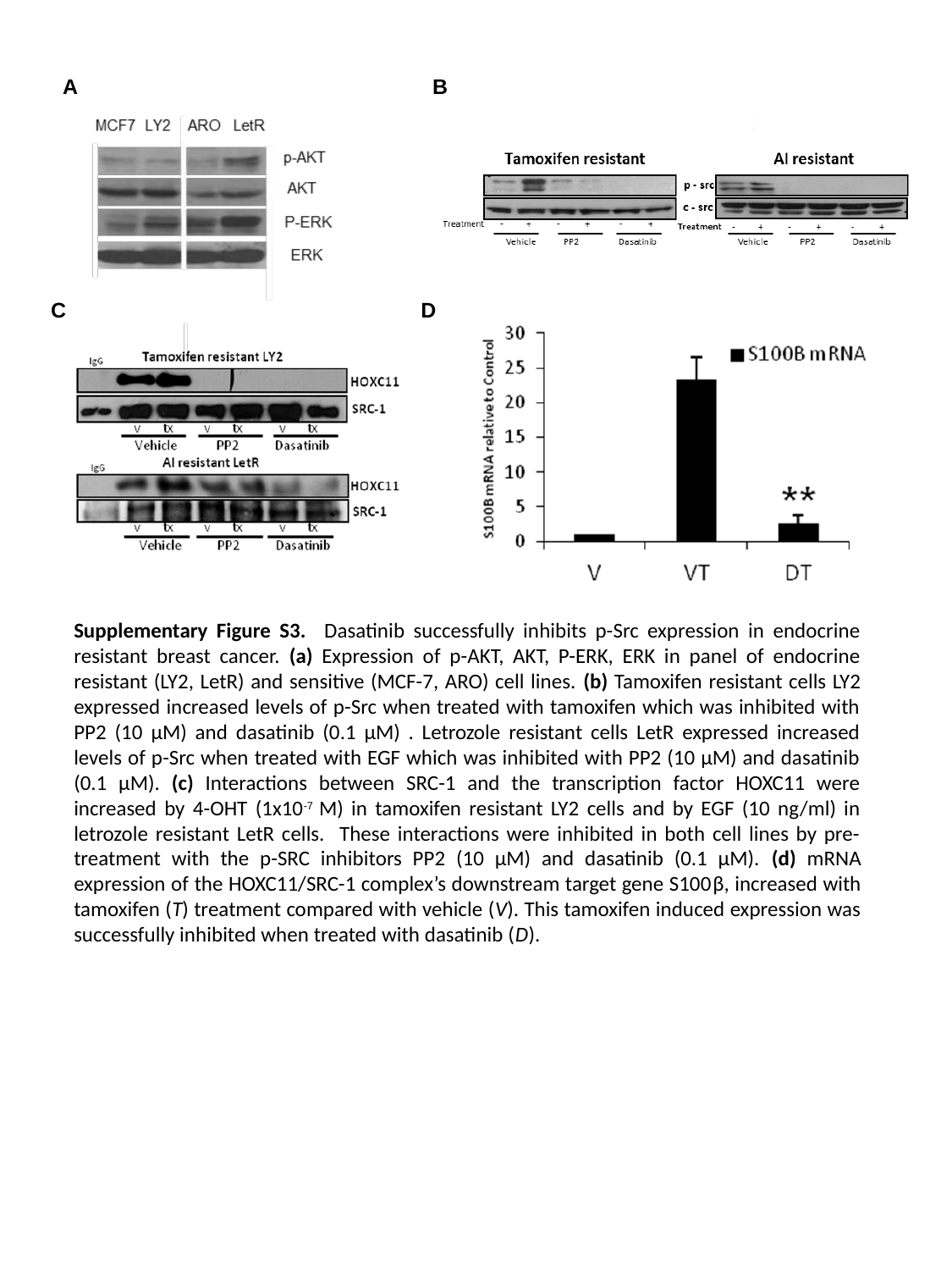

B
A
C
D
Supplementary Figure S3. Dasatinib successfully inhibits p-Src expression in endocrine resistant breast cancer. (a) Expression of p-AKT, AKT, P-ERK, ERK in panel of endocrine resistant (LY2, LetR) and sensitive (MCF-7, ARO) cell lines. (b) Tamoxifen resistant cells LY2 expressed increased levels of p-Src when treated with tamoxifen which was inhibited with PP2 (10 µM) and dasatinib (0.1 µM) . Letrozole resistant cells LetR expressed increased levels of p-Src when treated with EGF which was inhibited with PP2 (10 µM) and dasatinib (0.1 µM). (c) Interactions between SRC-1 and the transcription factor HOXC11 were increased by 4-OHT (1x10^-7^ M) in tamoxifen resistant LY2 cells and by EGF (10 ng/ml) in letrozole resistant LetR cells. These interactions were inhibited in both cell lines by pre-treatment with the p-SRC inhibitors PP2 (10 µM) and dasatinib (0.1 µM). (d) mRNA expression of the HOXC11/SRC-1 complex’s downstream target gene S100β, increased with tamoxifen (T) treatment compared with vehicle (V). This tamoxifen induced expression was successfully inhibited when treated with dasatinib (D).

## Slide 4
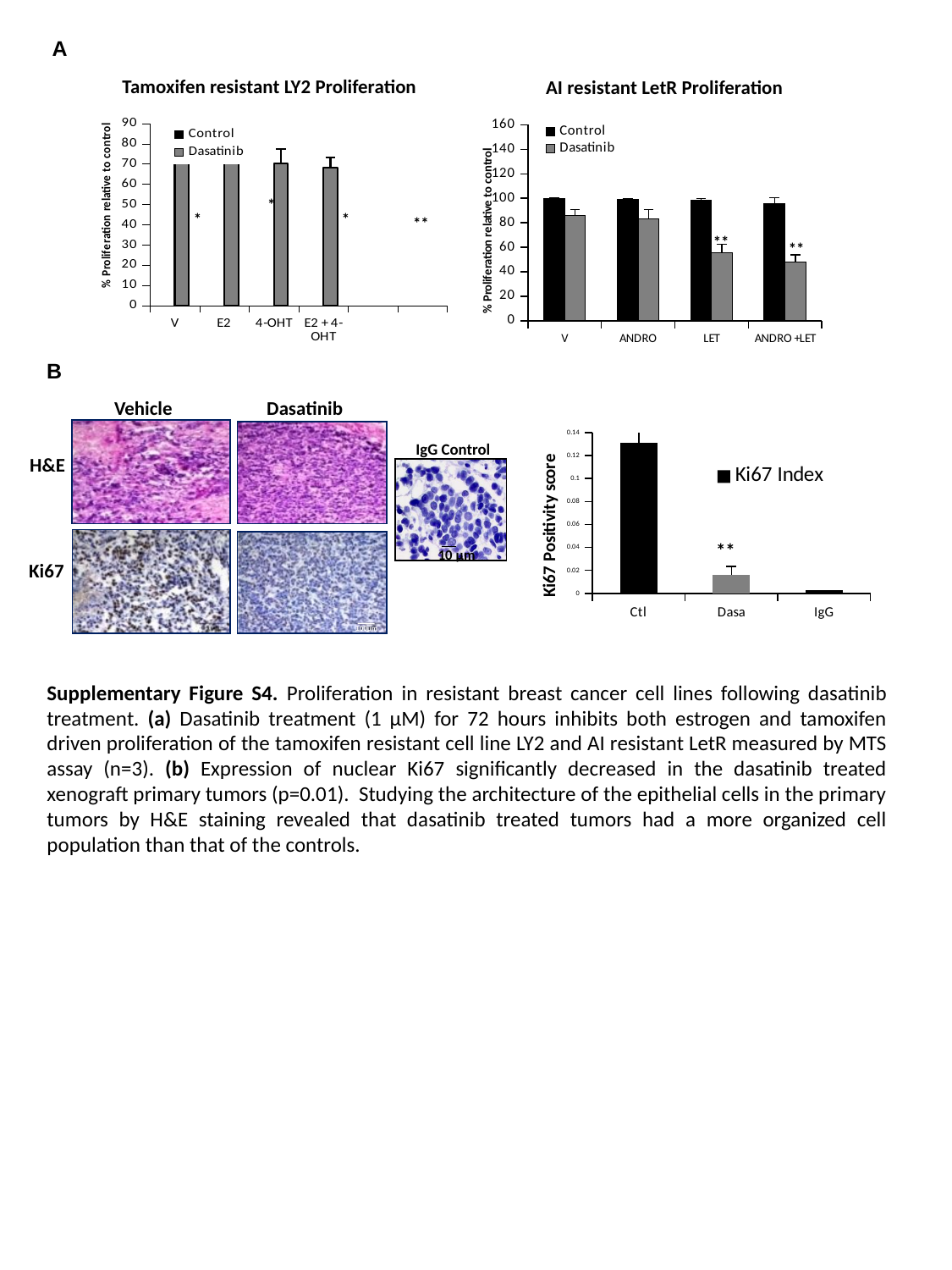

A
Tamoxifen resistant LY2 Proliferation
AI resistant LetR Proliferation
### Chart
| Category | | |
|---|---|---|
| V | 99.96030735762726 | 72.26822222618091 |
| E2 | 109.99871064498932 | 84.72036878145096 |
| 4-OHT | 117.40432123920672 | 70.21327012882878 |
| E2 + 4-OHT | 125.1866413117679 | 68.27851299504033 |
### Chart
| Category | | |
|---|---|---|
| V | 100.08556904200975 | 85.65586313045205 |
| ANDRO | 99.43677991807492 | 83.1619199081869 |
| LET | 98.54500189076292 | 55.75442144732289 |
| ANDRO +LET | 95.67394574986821 | 48.231321461711794 |*
*
*
**
**
**
B
Dasatinib
Vehicle
H&E
Ki67
### Chart
| Category | |
|---|---|
| Ctl | 0.1311393333333334 |
| Dasa | 0.015880000000000123 |
| IgG | 0.002560000000000049 |**
IgG Control
10 µm
100μm
Supplementary Figure S4. Proliferation in resistant breast cancer cell lines following dasatinib treatment. (a) Dasatinib treatment (1 µM) for 72 hours inhibits both estrogen and tamoxifen driven proliferation of the tamoxifen resistant cell line LY2 and AI resistant LetR measured by MTS assay (n=3). (b) Expression of nuclear Ki67 significantly decreased in the dasatinib treated xenograft primary tumors (p=0.01). Studying the architecture of the epithelial cells in the primary tumors by H&E staining revealed that dasatinib treated tumors had a more organized cell population than that of the controls.

## Slide 5
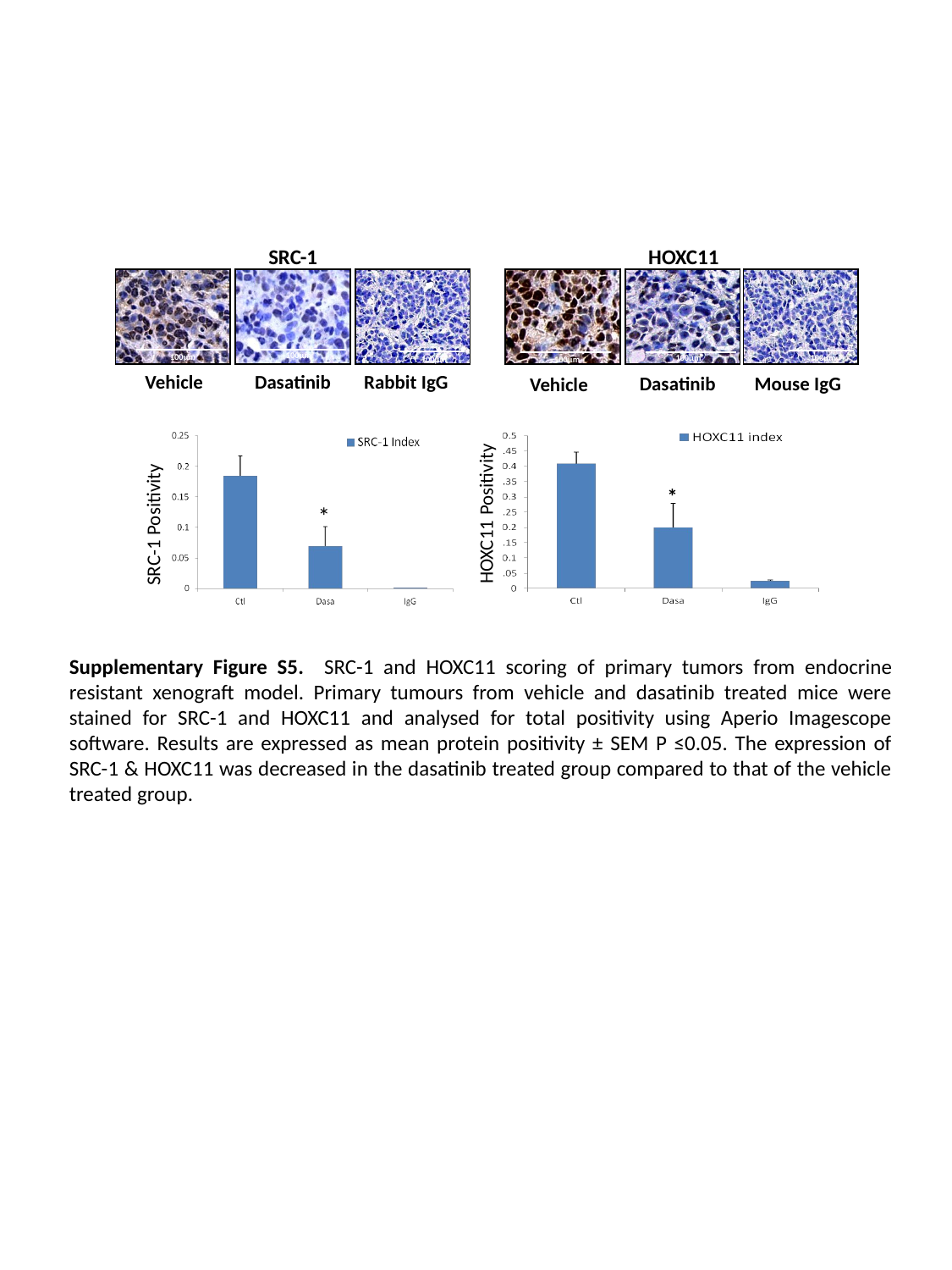

SRC-1
HOXC11
100µm
100µm
100µm
100µm
100µm
100µm
Vehicle
Dasatinib
Rabbit IgG
Mouse IgG
Dasatinib
Vehicle
*
*
HOXC11 Positivity
SRC-1 Positivity
Supplementary Figure S5. SRC-1 and HOXC11 scoring of primary tumors from endocrine resistant xenograft model. Primary tumours from vehicle and dasatinib treated mice were stained for SRC-1 and HOXC11 and analysed for total positivity using Aperio Imagescope software. Results are expressed as mean protein positivity ± SEM P ≤0.05. The expression of SRC-1 & HOXC11 was decreased in the dasatinib treated group compared to that of the vehicle treated group.

## Slide 6
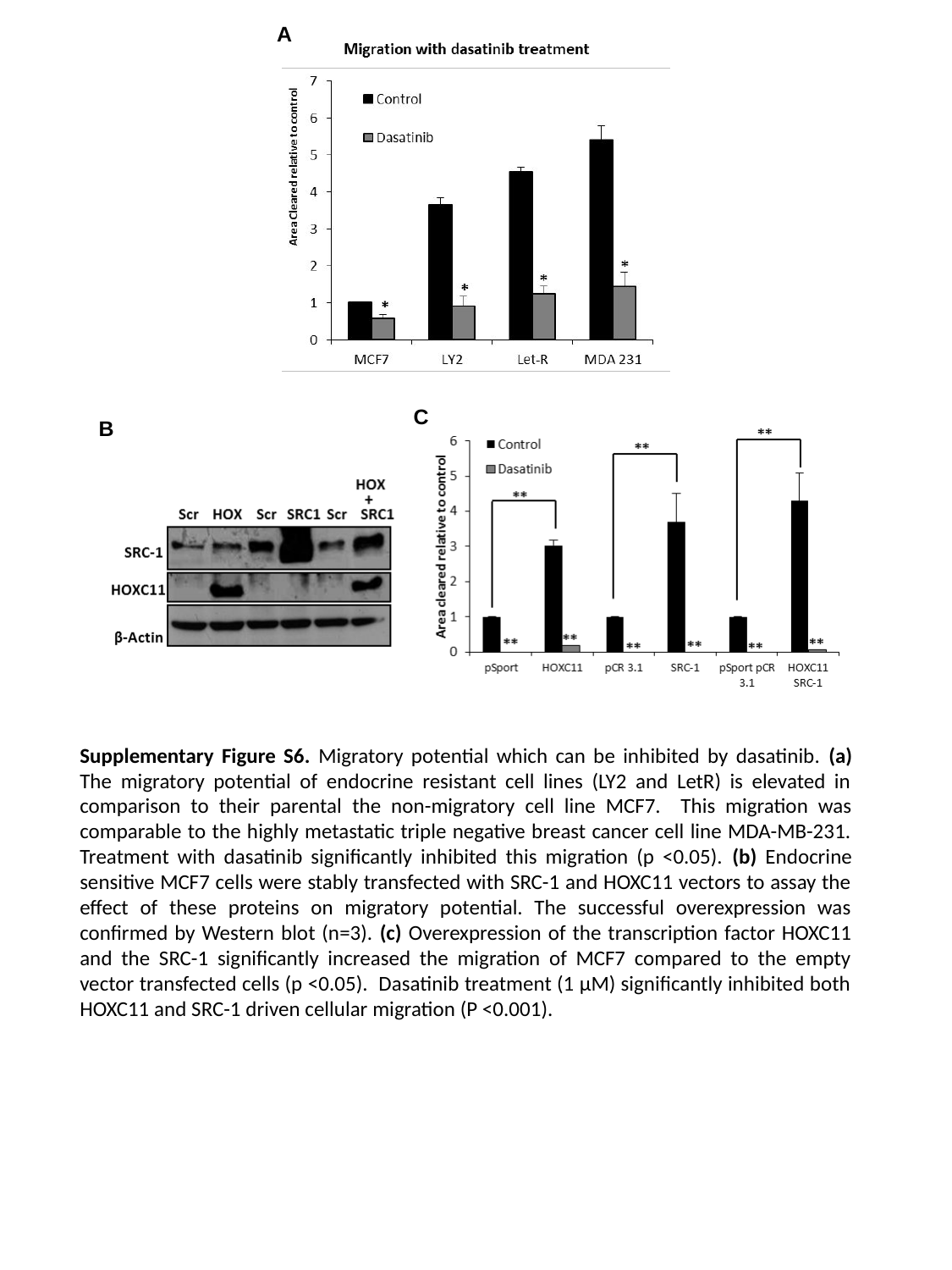

A
C
B
Supplementary Figure S6. Migratory potential which can be inhibited by dasatinib. (a) The migratory potential of endocrine resistant cell lines (LY2 and LetR) is elevated in comparison to their parental the non-migratory cell line MCF7. This migration was comparable to the highly metastatic triple negative breast cancer cell line MDA-MB-231. Treatment with dasatinib significantly inhibited this migration (p <0.05). (b) Endocrine sensitive MCF7 cells were stably transfected with SRC-1 and HOXC11 vectors to assay the effect of these proteins on migratory potential. The successful overexpression was confirmed by Western blot (n=3). (c) Overexpression of the transcription factor HOXC11 and the SRC-1 significantly increased the migration of MCF7 compared to the empty vector transfected cells (p <0.05). Dasatinib treatment (1 µM) significantly inhibited both HOXC11 and SRC-1 driven cellular migration (P <0.001).
