# Supplementary material for: S100β as a serum marker in endocrine resistant breast cancer
Source: BMC Med. 2017 Apr 12;15:79. doi: 10.1186/s12916-017-0836-2 (PMC5389184; doi:10.1186/s12916-017-0836-2)
Supplement: Supplementary file 2 — (A) S100β validation set (n = 76 ER-positive and ER-negative patients). (B) S100β validation set (n = 59 ER-positive patients). Association of S100β status with clinicopathological variables and disease recurrence using Fisher’s exact test. Table S2. Pre-operative and post-operative S100β serum levels in 55 ER-positive patients. Association of S100β status with clinicopathological variables using Fisher’s exact test. Table S3. S100β tissue expression in matched primary and metastatic tissue from ER-positive patients. Table S4. Patient details from explant study. Explant endocrine resistant tumor tissue (n = 2) was treated with AI therapy (letrozole) in the presence and absence of dasatinib. (PPTX 93 kb) [file 12916_2017_836_MOESM2_ESM.pptx]

## Slide 1
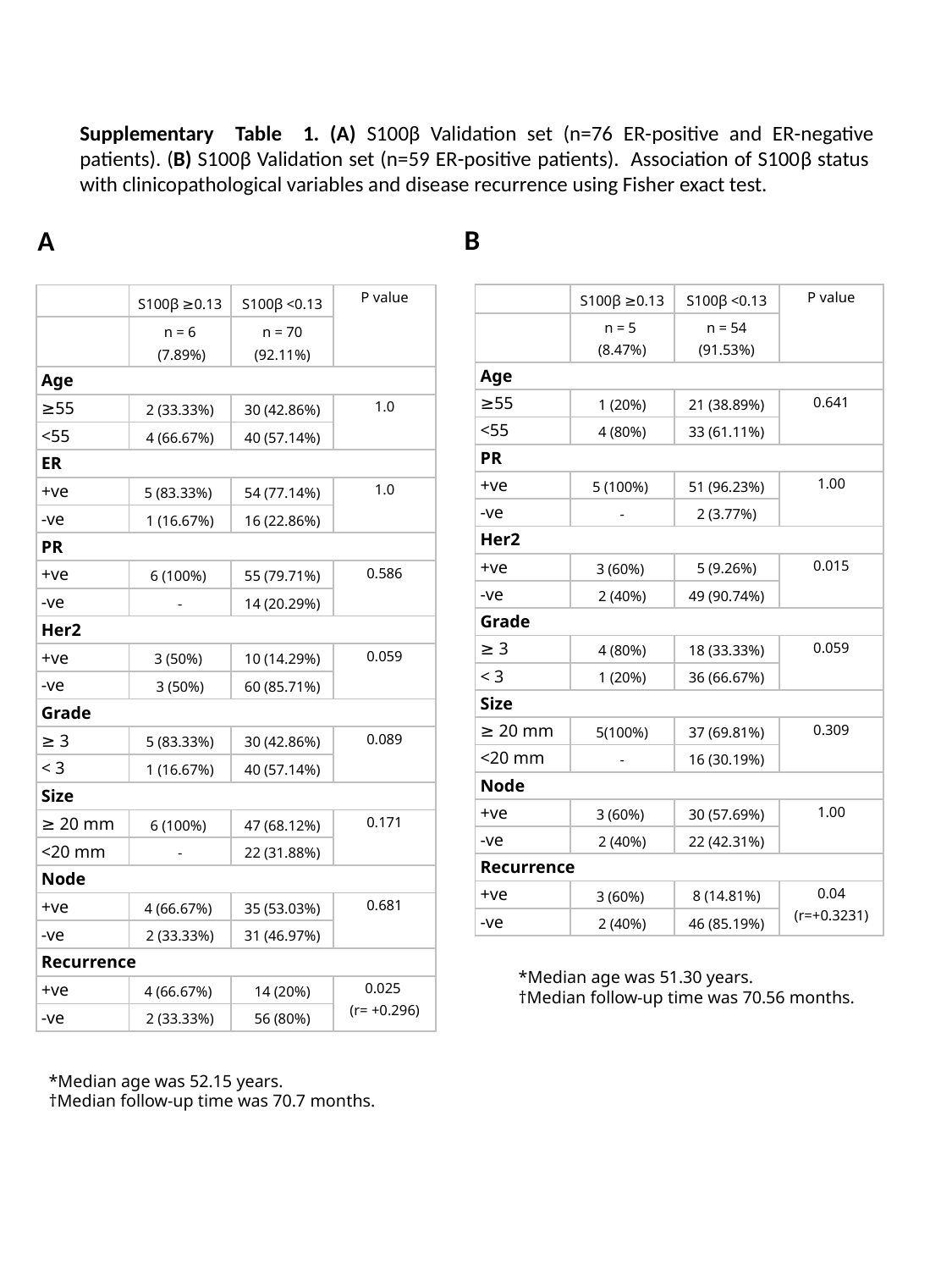

Supplementary Table 1. (A) S100β Validation set (n=76 ER-positive and ER-negative patients). (B) S100β Validation set (n=59 ER-positive patients). Association of S100β status with clinicopathological variables and disease recurrence using Fisher exact test.
B
A
| | S100β ≥0.13 | S100β <0.13 | P value |
| --- | --- | --- | --- |
| | n = 5 (8.47%) | n = 54 (91.53%) | |
| Age | | | |
| ≥55 | 1 (20%) | 21 (38.89%) | 0.641 |
| <55 | 4 (80%) | 33 (61.11%) | |
| PR | | | |
| +ve | 5 (100%) | 51 (96.23%) | 1.00 |
| -ve | - | 2 (3.77%) | |
| Her2 | | | |
| +ve | 3 (60%) | 5 (9.26%) | 0.015 |
| -ve | 2 (40%) | 49 (90.74%) | |
| Grade | | | |
| ≥ 3 | 4 (80%) | 18 (33.33%) | 0.059 |
| < 3 | 1 (20%) | 36 (66.67%) | |
| Size | | | |
| ≥ 20 mm | 5(100%) | 37 (69.81%) | 0.309 |
| <20 mm | - | 16 (30.19%) | |
| Node | | | |
| +ve | 3 (60%) | 30 (57.69%) | 1.00 |
| -ve | 2 (40%) | 22 (42.31%) | |
| Recurrence | | | |
| +ve | 3 (60%) | 8 (14.81%) | 0.04 (r=+0.3231) |
| -ve | 2 (40%) | 46 (85.19%) | |
| | S100β ≥0.13 | S100β <0.13 | P value |
| --- | --- | --- | --- |
| | n = 6 (7.89%) | n = 70 (92.11%) | |
| Age | | | |
| ≥55 | 2 (33.33%) | 30 (42.86%) | 1.0 |
| <55 | 4 (66.67%) | 40 (57.14%) | |
| ER | | | |
| +ve | 5 (83.33%) | 54 (77.14%) | 1.0 |
| -ve | 1 (16.67%) | 16 (22.86%) | |
| PR | | | |
| +ve | 6 (100%) | 55 (79.71%) | 0.586 |
| -ve | - | 14 (20.29%) | |
| Her2 | | | |
| +ve | 3 (50%) | 10 (14.29%) | 0.059 |
| -ve | 3 (50%) | 60 (85.71%) | |
| Grade | | | |
| ≥ 3 | 5 (83.33%) | 30 (42.86%) | 0.089 |
| < 3 | 1 (16.67%) | 40 (57.14%) | |
| Size | | | |
| ≥ 20 mm | 6 (100%) | 47 (68.12%) | 0.171 |
| <20 mm | - | 22 (31.88%) | |
| Node | | | |
| +ve | 4 (66.67%) | 35 (53.03%) | 0.681 |
| -ve | 2 (33.33%) | 31 (46.97%) | |
| Recurrence | | | |
| +ve | 4 (66.67%) | 14 (20%) | 0.025 (r= +0.296) |
| -ve | 2 (33.33%) | 56 (80%) | |
*Median age was 51.30 years.
†Median follow-up time was 70.56 months.
*Median age was 52.15 years.
†Median follow-up time was 70.7 months.

## Slide 2
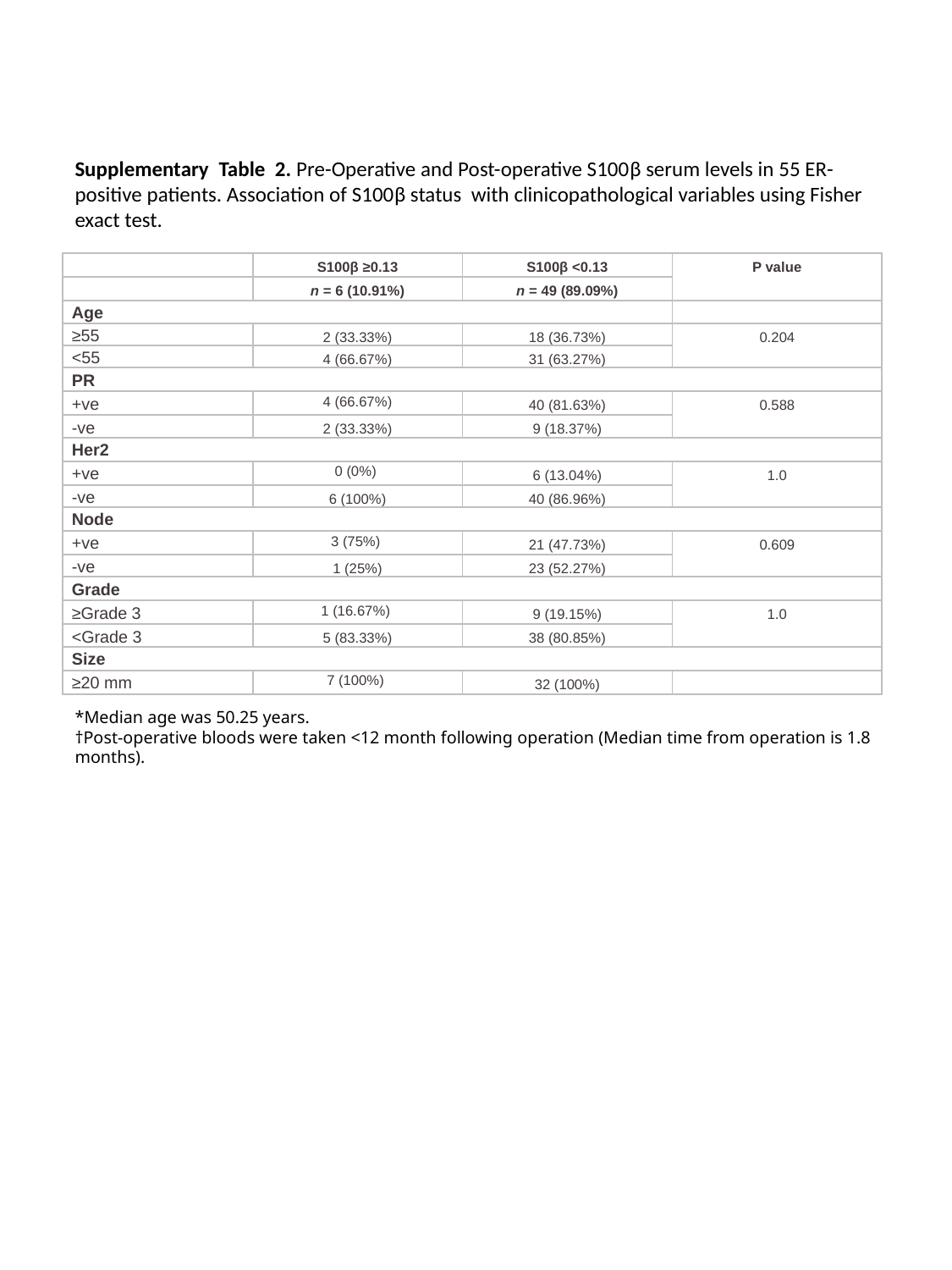

Supplementary Table 2. Pre-Operative and Post-operative S100β serum levels in 55 ER-positive patients. Association of S100β status with clinicopathological variables using Fisher exact test.
| | S100β ≥0.13 | S100β <0.13 | P value |
| --- | --- | --- | --- |
| | n = 6 (10.91%) | n = 49 (89.09%) | |
| Age | | | |
| ≥55 | 2 (33.33%) | 18 (36.73%) | 0.204 |
| <55 | 4 (66.67%) | 31 (63.27%) | |
| PR | | | |
| +ve | 4 (66.67%) | 40 (81.63%) | 0.588 |
| -ve | 2 (33.33%) | 9 (18.37%) | |
| Her2 | | | |
| +ve | 0 (0%) | 6 (13.04%) | 1.0 |
| -ve | 6 (100%) | 40 (86.96%) | |
| Node | | | |
| +ve | 3 (75%) | 21 (47.73%) | 0.609 |
| -ve | 1 (25%) | 23 (52.27%) | |
| Grade | | | |
| ≥Grade 3 | 1 (16.67%) | 9 (19.15%) | 1.0 |
| <Grade 3 | 5 (83.33%) | 38 (80.85%) | |
| Size | | | |
| ≥20 mm | 7 (100%) | 32 (100%) | |
*Median age was 50.25 years.
†Post-operative bloods were taken <12 month following operation (Median time from operation is 1.8 months).

## Slide 3
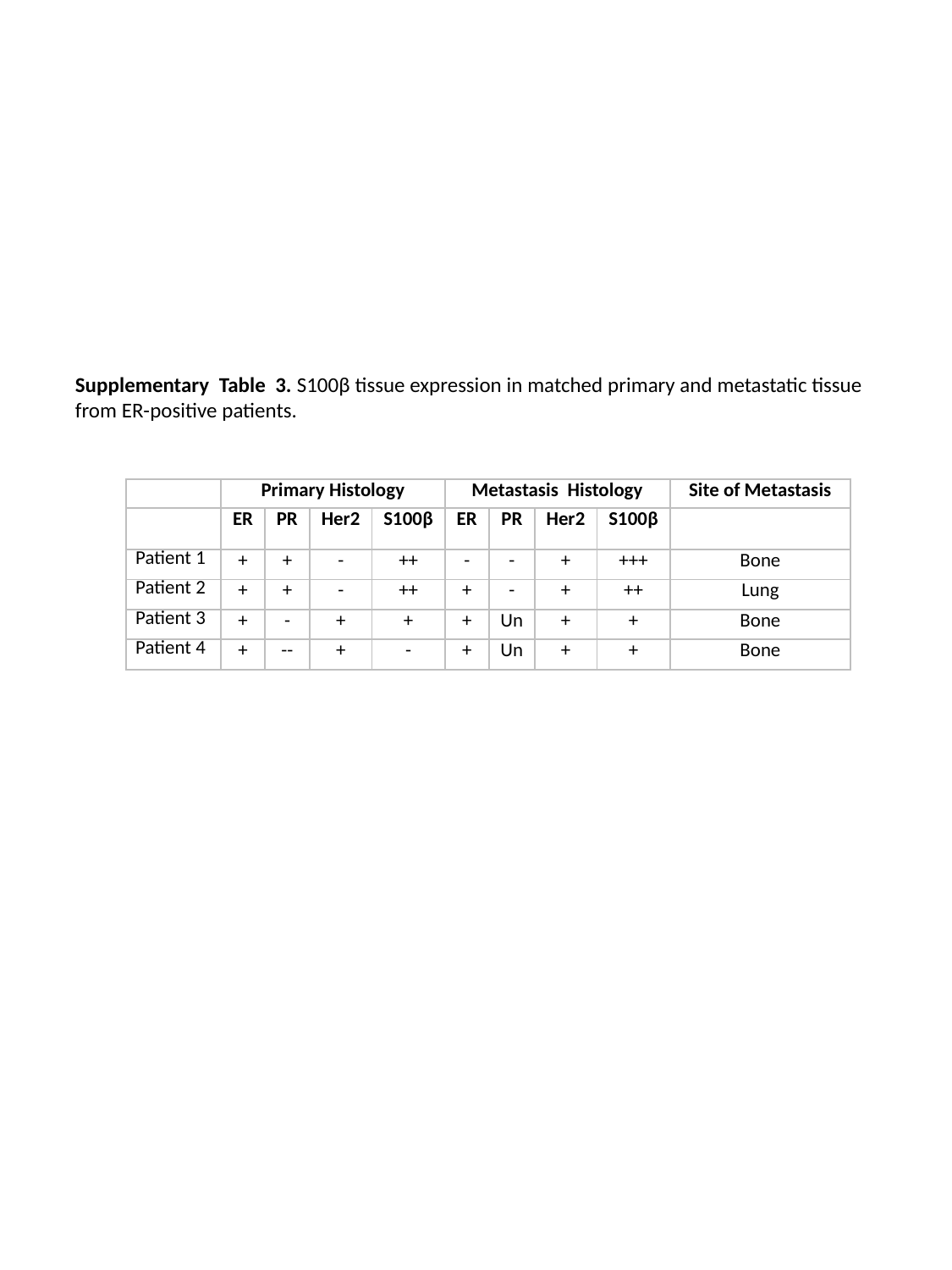

Supplementary Table 3. S100β tissue expression in matched primary and metastatic tissue from ER-positive patients.
| | Primary Histology | | | | Metastasis Histology | | | | Site of Metastasis |
| --- | --- | --- | --- | --- | --- | --- | --- | --- | --- |
| | ER | PR | Her2 | S100β | ER | PR | Her2 | S100β | |
| Patient 1 | + | + | - | ++ | - | - | + | +++ | Bone |
| Patient 2 | + | + | - | ++ | + | - | + | ++ | Lung |
| Patient 3 | + | - | + | + | + | Un | + | + | Bone |
| Patient 4 | + | -- | + | - | + | Un | + | + | Bone |

## Slide 4
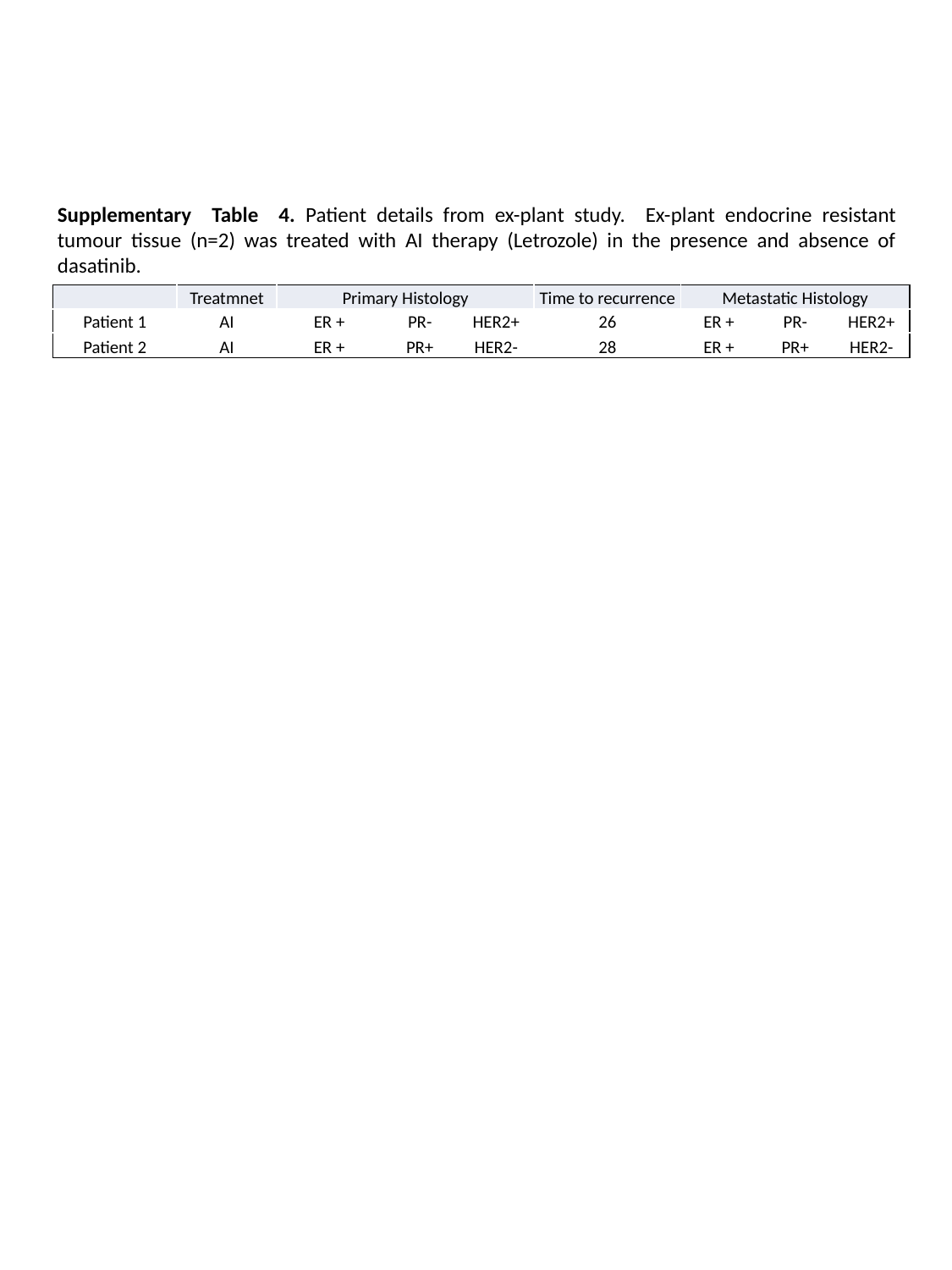

Supplementary Table 4. Patient details from ex-plant study. Ex-plant endocrine resistant tumour tissue (n=2) was treated with AI therapy (Letrozole) in the presence and absence of dasatinib.
| | Treatmnet | Primary Histology | | | Time to recurrence | Metastatic Histology | | |
| --- | --- | --- | --- | --- | --- | --- | --- | --- |
| Patient 1 | AI | ER + | PR- | HER2+ | 26 | ER + | PR- | HER2+ |
| Patient 2 | AI | ER + | PR+ | HER2- | 28 | ER + | PR+ | HER2- |
